# Prolactin and DNA damage trigger an anti-breast cancer cell immune response

**DOI:** 10.3389/fendo.2025.1586062

**Published:** 2025-09-23

**Authors:** Ödül Karayazi Atici, Nayantara Govindrajan, Isbel Lopetegui-González, Constance A. M. Finney, Carrie S. Shemanko

**Affiliations:** ^1^ Department of Biological Sciences, University of Calgary, Calgary, AB, Canada; ^2^ Host Parasite Interactions Network, University of Calgary, Calgary, AB, Canada; ^3^ Arnie Charbonneau Cancer Institute, University of Calgary, Calgary, AB, Canada

**Keywords:** prolactin, DNA damage response, natural killer cells, breast cancer, xenograft

## Abstract

**Introduction:**

The role of prolactin (PRL) in breast cancer and its role within the context of the tumor microenvironment are not well understood. In our previous study, we demonstrated a cross-talk between the ataxia telangiectasia-mutated (ATM) DNA damage response pathway and the PRL-Janus-kinase-2 (JAK2)-signal transducer and activator of transcription-5 (STAT5)-heat shock protein-90 (HSP90) pathway. Here we investigated the role of PRL in tumor initiation and the effect of DNA damage.

**Methods:**

We used an *in vivo* model to assess the ability of breast cancer cells to initiate orthotopic xenograft tumor formation after DNA damage. Breast cancer cells engineered to secrete human PRL were treated with the DNA damaging agent doxorubicin and injected into the mammary fat pad of immune-deficient severe combined immunodeficiency disease (SCID) mice.

**Results:**

Doxorubicin and PRL combination increased the tumor latency, although PRL secretion alone did not change the tumor latency compared to the controls. Depletion of glycolipid asialo ganglioside-GM1-positive immune cells using anti-asialo GM1 antibody resulted in faster tumor formation only in the PRL-secreting breast cancer cells that were pre-treated with doxorubicin. Additionally, doxorubicin plus the PRL treatment of breast cancer cells was shown *in vitro* to attract cytotoxic NK cells compared to the controls, and this was dependent on the PRLR.

**Discussion:**

These results demonstrate that combined breast cancer cell DNA damage and PRL exposure results in the anti-tumor cell activity of asialo-GM1-positive immune cells.

## 1 Introduction

Prolactin (PRL) is a peptide hormone that promotes the proliferation, differentiation, survival, and motility of mammary epithelial and mammary or breast tumor cells upon binding to its receptor (PRLR) (1). Although confirmed as a lactogenic hormone (2), its interaction with the PRLR is implicated in the progression and metastasis of breast cancer (3–5). PRL is secreted from the pituitary gland and extra-pituitary sites in humans where it behaves as a paracrine/autocrine signaling molecule (6, 7).

Previous studies demonstrated that endocrine and autocrine PRL signaling in murine models contribute to increased mammary tumor formation (8–12), though there are some observations that PRL may have the opposite effect (13). It was reported that monomeric complexes of long PRLR isoforms promoted mammary differentiation, while the intermediate and long PRLR heteromeric complexes promoted tumorigenesis (14). These studies highlight the complexity of PRL signaling and the need to delineate the conditions by which PRL has differing effects on breast cancer.

PRL has been demonstrated to have a role in the cytotoxic resistance of breast cancer to a variety of chemotherapy drugs *in vitro* (15, 16). We demonstrated that PRL increases the viability of breast cancer cells treated with DNA-damaging agents, doxorubicin, or etoposide and that it was specific to the PRLR. Our studies confirmed that doxorubicin and etoposide induced DNA damage and activates the DNA damage response protein, ataxia telangiectasia-mutated (ATM). The molecular mechanism established in the study showed that ATM was required for the PRL-Janus-kinase-2 (JAK2)-signal transducer and activator of transcription-5 (STAT5)-heat shock protein-90 (HSP90) pathway to mediate this resistance (15).

Here we used an orthotopic model to investigate the role of autocrine PRL in breast tumor progression in the context of DNA damage induced by doxorubicin (15) and the presence of immune cells. Severe combined immunodeficiency disease (SCID) mice have severe deficiency in T and B cells; however, they have active natural killer (NK) cells, which sometimes limit the growth of human xenografts (17, 18). The anti-asialo-GM1 antibody was developed (19) to deplete the activity of NK cells and other asialo-GM1-positive immune cells. We report that autocrine PRL supports the initiation and growth of xenografts but that the combination of PRL and the DNA damage response triggers an attack from asialo-GM1-positive immune cells in SCID mice.

## 2 Materials and methods

### 2.1 Materials

Doxorubicin (Sigma-Aldrich Canada Co., Oakville, ON, Canada), 10 mg, was dissolved in sterile DMSO. Human recombinant PRL was purchased from A.F. Parlow, National Hormone and Pituitary Program, CA, USA.

### 2.2 Antibodies

A list of antibodies is provided in the supplement.

### 2.3 Cell culture and cell lines

The human cell lines used in this study (MCF7, SKBR3, and NK92MI) were obtained and authenticated from American Type Culture Collection (ATCC) and were used within 6 months when revived from storage. Mycoplasma testing was routinely performed using pan-species primers and PCR. Breast cancer cell lines were maintained in DMEM (Invitrogen, Burlington, ON, Canada), with 10% fetal bovine serum (FBS) (PAA Laboratories Inc., Etobicoke, ON, Canada). NK9MI cells were maintained in Minimum Essential Medium Eagle (Millipore Sigma, Burlington, ON, Canada) with 12.5% FBS, 12.5 horse serum (Invitrogen) (ATCC).

### 2.4 Cell treatments

Breast cancer cells, 1 × 10^6^, were plated into 10-cm cell culture plates and on the following day treated or not with human recombinant PRL (25 ng/mL) for 24 h. On the next day, the cells were treated or not with doxorubicin (1 uM) for 2 h and recovered for 48 h.

### 2.5 Creation of syngeneic lines

The human PRL pcDNA3.1/Zeo(+) mammalian expression vector (plasmid, a gift from Dr. Vincent Goffin, Inserm and University Paris Descartes, Paris, France) (9) or empty pcDNA3.1/Zeo(+) plasmid was transfected into MCF7 cells using polyethyleminine (PEI) MW25K (Polyscience Inc., Warrington, PA, USA). The stably transfected cells carrying the Zeocin antibiotic resistance gene (Sh ble) were selected with and maintained in Zeocin (800 mg/mL) (Invivogen, San Diego, CA, USA). Cells carrying the empty vector were confirmed by PCR (see the method).

PRLR knockout (KO) SKBR3 cells were created using clustered regularly interspaced palindromic repeats (CRISPR)/CRISPR-associated protein 9 (Cas9) genomic engineering, and the PRLR KO was confirmed by genome sequencing (Center for Genome Engineering, University of Calgary). Wild-type (WT) and CRISPR Control (CC) (single-cell clone controls) cells were used as controls for two independent PRLR KO SKBR3 colonies (SKBR3KO1 and SKBR3KO2).

### 2.6 Cell viability assay

Alamar blue cell viability reagent (Invitrogen, Burlington, ON, Canada) was used according to the manufacturer’s instructions to determine the cellular proliferation and viability of the cells. The cell treatments and fluorescence intensity normalization were previously described (15).

### 2.7 Nuclear lysate extract

Nuclear lysate extract was used to identify phosphorylated-STAT5 (p-STAT5), STAT5 as previously described (20) and TATA-Binding protein (TBP). Cells, 1 × 10^6^, were plated in 10-cm plates and, on the next day, treated for 30 min with human recombinant PRL. Immunoblotting for STAT5, p-STAT5 and growth factor receptor-bound protein 2 (GRB2) was performed as previously described (15). GRB2 was used as the loading control.

### 2.8 Soluble senescence-associated β-galactosidase activity assay

Breast cancer cells were plated into 10-cm cell culture plates in 1 × 10^6^ cell number and on the following day treated or not with human recombinant PRL (25 ng/mL) for 24 h. On the next day, the cells were treated or not with doxorubicin (1 uM) or vehicle control for 2 h and recovered for 6 days to induce and detect senescence.

To prevent confluency-induced senescence, the cells were split once if required. The cells were collected and centrifuged, the pellet was washed in 1X PBS and resuspended in 0.2 M phosphate buffer at pH 6.0 (87.7 mL of 0.4 M sodium phosphate monobasic, 12.3 mL of 0.4 M sodium phosphate dibasic heptahydrate, 100 mL ddH_2_O), subjected to three cycles of freeze and thaw. The cells were centrifuged, and protein concentration in the supernatant was measured with BioRad protein assay; equal protein values were used for all of the experimental groups. The protein samples were incubated in a senescence assay buffer (21) containing 2 mM MgCl_2_, 100 mM b-mercaptoethanol, 1.3 mg/mL ONPG (from previously prepared 4 mg/mL 2-nitrophenyl b-D-galactopyranoside in 0.2 M phosphate buffer pH 6.0), and 0.2 M phosphate buffer pH 6.0 for 4 to 9 h at 37°C. The reaction was stopped with 1 M Na_2_CO_3_. Optical densities (420 nm Spectramax M4 Microplate Reader) were averaged for internal and experimental replicates, and standard deviations were calculated.

### 2.9 Calcein-AM assay

Calcein-AM assay was used to test killer cell cytotoxicity (22). Calcein-AM (Invitrogen, ON, Canada) work solution (1 mg/mL in DMSO) was prepared in serum-free DMEM at 2 ug/mL concentration. Following the cell treatments, the cells were collected within media and centrifuged. Following counting, the cells were resuspended in Calcein-AM-containing media (1 × 10^6^ cells/mL) and incubated for 30 min at 37 °C. Calcein-AM-stained breast cancer cells were washed twice with 1X PBS and co-cultured with NK92MI human NK cells for 24 h s at 1:1 or 1:10 target/effector ratio. The fluorescence was read from CM at wavelength excitation: 485 nm, emission: 535 nm. Calcein-AM-stained breast cancer cells without NK cells were used as a control for spontaneous release, and the breast cancer cells treated for 5 min with 1% Triton X-100 were used as maximum release control. The %lysis was calculated based on the calculation below.


$$\%\text{Lysis}=\frac{\left(\text{Test release}-\text{Spontaneous Release}\right)}{\left(\text{Max Release}-\text{Spontaneous Release}\right)}\times 100$$

### 2.10 Xenograft animal models

All animal procedures were carried out strictly following the Canadian Council for Animal Care guidelines and ethics approval from the University of Calgary Life and Environmental Sciences Animal Care Committee.

To stimulate tumor growth with estrogen receptor-positive breast cancer cells in mice (23), estrogen (17b-estradiol) pellets (0.72 mg/pellet, 60-day release, cat. no. SE-121, Innovative Research of America, Sarasota, FL, USA) (24) were inserted subcutaneously into 9-week-old Fox Chase SCID female mice (strain 236) (Charles River Laboratories, Montreal, QC, Canada). The cell pellets were washed and resuspended in cold PBS and Cultrex BME (Cedarlane, Burlington, ON, Canada) mixture and injected into the fourth mammary fat pad of mice. Contralateral mammary fat pads were injected with PBS and Cultrex BME mixture. To deplete NK cell activity in SCID mice, 20 µL of the 1.1. mg of anti-mouse asialo-GM1 (Cedarlane, Burlington, ON, Canada) or control serum (ImmunoReagents, Raleigh, NC, USA) was injected intraperitoneally every 3 to 4 days for the duration of the experiment, following the titration data from the manufacturer. The tumor volumes were calculated as follows: [*V* = (*W*2 × *L*)/2], where *V* is tumor volume, *W* is tumor width, and *L* is tumor length. In tumor latency studies with or without the use of anti-asialo antibody, *n* = 5 mice or *n* = 15 were used; in flow cytometry studies, *n* = 3 or *n* = 6 mice were used following the initial experimental optimization.

### 2.11 Mammary gland digestion

Mammary glands were resected, minced, and incubated in a dissociation buffer containing gentle collagenase/hyaluronidase (StemCell Technologies, Vancouver, BC, Canada) and DMEM/F12 (Gibco) with 10% FBS. The erythrocytes were lysed and removed using a 0.8% ammonium chloride solution in FACS buffer containing 1X PBS and 5% FBS. Following centrifugation, the pellet was resuspended in 0.25% trypsin and deactivated by FACS buffer and centrifuged, followed by Dispase (5 U/mL, StemCell Technologies) and DNase I solution (1 mg/mL, StemCell Technologies) and filtered using a 40-um strainer. The cells were directly used for flow cytometry analysis or fixed using 2% PFA after being stained with fixable viability stain.

### 2.12 Flow cytometry

The flow cytometry experimental design details are described in the methods. The cells, 1 × 10^6^, were washed once and resuspended in sodium azide and protein-free Dulbecco’s phosphate-buffered saline (1X DPBS) without FBS. Then, 0.3 µL of BD Horizon™ Fixable Viability Stain eFluor450-A Stock Solution was added per 1 mL of cell suspension and vortexed immediately. After 15 min of incubation in the dark at room temperature, the cells were washed twice with FACS buffer and stained directly with antibodies or fixed in 2% PFA. The cells were incubated with Mouse Fc Block purified anti-mouse CD16/CD32 mAb (BD Horizon) at 1 ug/million cells in 100 uL FACS buffer for 5 min at 4°C. Master mixes containing fluorescent-labeled antibodies, Florescence Minus One (FMO) controls, IgG, and secondary antibody controls were prepared in FACS buffer; the samples were incubated for 30 min in the dark. The washed samples were transferred to FACS tubes for immediate analysis.

The list of antibodies is provided in methods and used according to the manufacturer’s protocol. FlowJo software was used for the analysis of all FCS files.

### 2.13 Gating strategy

Single cells were gated based on forward and side scatter patterns. Cells that were negative for fixable viability dye FVD eFluor 450 were selected as live cells. The immune population was chosen *in vivo* by gating for CD45-AF700-positive cells. Within this CD45-positive population, NK cells were identified as DX5-FITC+, mature NK cells were identified as NKP46-PerCP-efluor710+ (25, 26), and macrophages were identified as F4/80-BV421-A+ (Wilson et al., 2022). The values were evaluated in injected and contralateral glands, normalized to the uninjected gland, and presented as relative percentages of CD45+DX5+ cells, NKp46+ cells within CD45+DX5+cells, or CD45+F4/80+cells. The gates for each dye were adjusted based on single color controls, FMO controls, and isotype staining for relevant antibodies.

### 2.14 Statistics

Statistical significance was tested with a paired or unpaired Student’s *t*-test; alternatively, analysis of variance (ANOVA) was used with post-testing for multiple comparisons. Log-rank (Mantel–Cox) and Gehan–Breslow Wilcoxon statistical analyses were used to determine differences in tumor latency. One-way ANOVA followed by Mann–Whitney *U*-test was used to analyze tumor volumes. The results were considered significant when the *P*-value was lower than 0.05 (*P*<.05).

## 3 Results

### 3.1 Preparation and validation of a PRL-secreting MCF7 cell line

In order to create a xenograft model with a consistent supply of human PRL for human breast cancer cells, MCF7 cells were stably transfected with a human PRL expression (hPRL) plasmid or the empty vector (EV). The cellular PRL levels were evaluated from whole cell extracts (**Supplementary Figure S1A**), and PRL secretion was evaluated from conditioned media (CM) using western blot (**Supplementary Figure S1B**). Colonies 1 and 5 were pooled (MCF7hPRL). The MCF7EV control line was confirmed by the amplification of the vector antibiotic (zeocin) resistance gene, Sh ble, in stable lines (**Supplementary Figure S1C**). The autocrine PRL secretion was evaluated over 7 days from CM without refreshing media, using an ELISA assay. MCF7hPRL cells secreted 24 ng/mL of PRL daily. A negligible amount of PRL secretion was detected from MCF7 and MCF7EV cells (**Supplementary Figure S1D**).

We examined phosphorylated-STAT5 (p-STAT5) as a read-out of PRL-JAK2-STAT5 pathway activation to confirm that the autocrine PRL successfully activates the PRLR. The MCF7hPRL cell line had levels of p-STAT5 similar with the two control cell lines treated with 25 ng/mL recombinant PRL (**Supplementary Figure S1E**), consistent with their calculated daily PRL secretion. Therefore, the MCF7hPRL cell line was confirmed to secrete PRL, and the secreted PRL activates STAT5 as a readout of PRLR activation.

### 3.2 Autocrine PRL causes cellular reduced sensitivity to doxorubicin

The effect of doxorubicin on PRL secretion was evaluated from CM by an ELISA assay from MCF7, MCF7EV, and MCF7hPRL cells following doxorubicin treatment (1 uM). Doxorubicin treatment did not affect PRL secretion from MCF7 or MCFEV cells, and it does appear to increase PRL secretion from MCF7hPRL cells on days 1 and 2, although the effect was not sustained (**Supplementary Figure S1D**).

We examined the PRLR levels following doxorubicin treatment, and our results confirmed that doxorubicin did not affect the PRLR levels in any of the MCF7 lines (**Supplementary Figure S1F**). The MCF7EV line has the highest level of PRLR compared to the parental or MCF7hPRL line (**Supplementary Figure S1F**).

We performed cell viability analysis to test if autocrine PRL increased the viability of doxorubicin-treated breast cancer cells, similar to recombinant PRL, as we have observed (15). The MCF7hPRL cells showed significant reduced sensitivity to doxorubicin at 3, 4, 5, and 6 uM concentrations (**Supplementary Figure S1G**) when compared to MCF7EV treatments. This result confirms that autocrine PRL also increases cellular viability against the DNA-damaging agent, doxorubicin.

### 3.3 Autocrine PRL delays tumor latency in the presence of DNA damage

A novel orthotopic model of breast cancer was used to investigate the role of autocrine PRL and the DNA damage response on tumorigenicity and tumor volume. MCF7hPRL or control cells were treated with the DNA-damaging agent, doxorubicin (1 uM), at conditions demonstrated to activate ATM in our previous study (15), and allowed to recover for 48 h before injection into the number 4 mammary fat pad of SCID mice. This model examines the ability of cells to form a tumor after chemotherapeutic treatment and test the effect of autocrine PRL on tumor initiation in a recurrence-style model (15).

**Figure 1:**
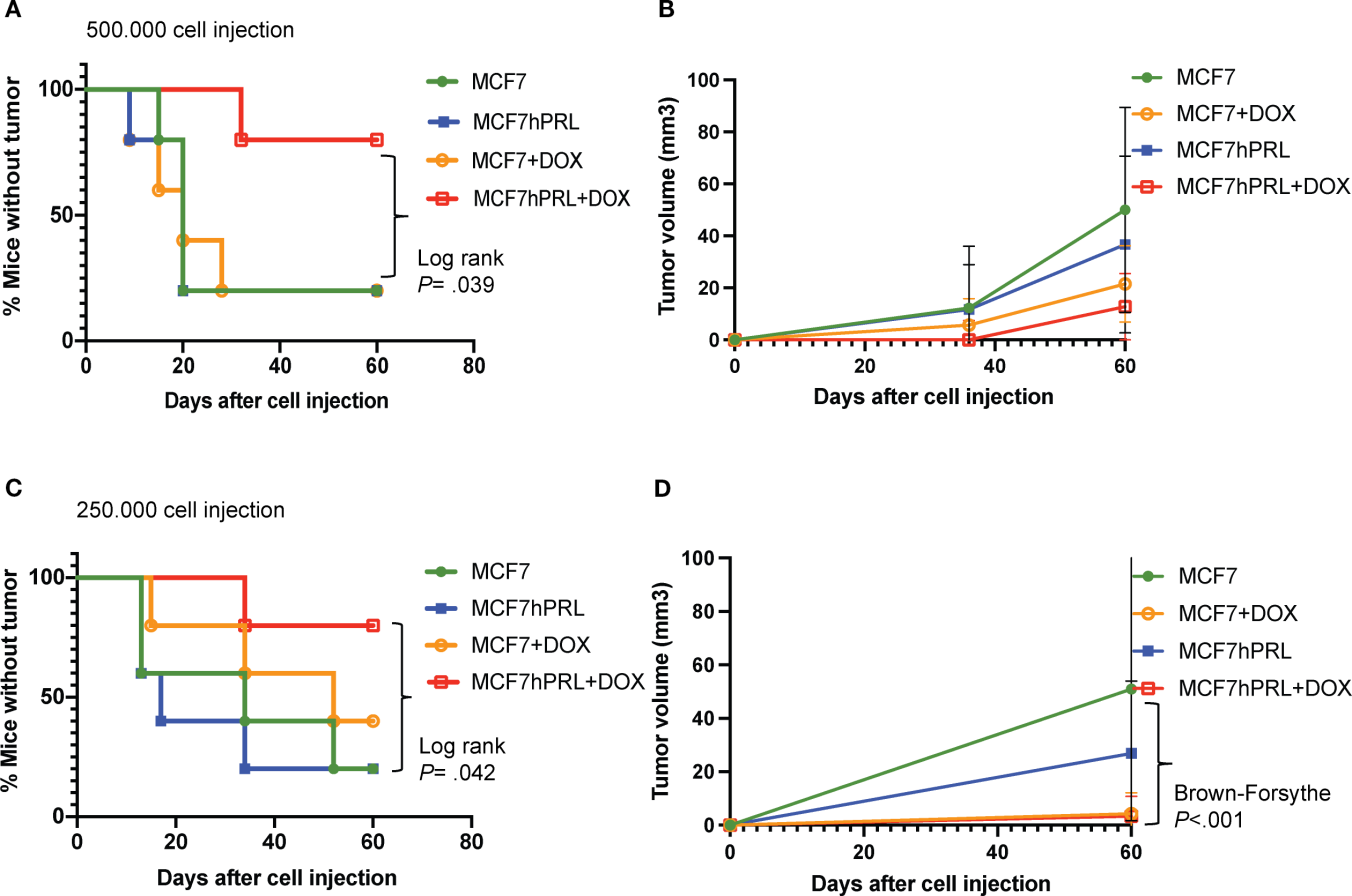
Autocrine PRL delays tumor latency in the presence of DNA damage in SCID mice. **(A)** Tumor latency in SCID mice after an injection of 500,000 MCF7 or MCF7hPRL cells -/+ doxorubicin over 60 days. **(B)** Comparison of accumulated tumor volumes between treatment groups over 60 days. **(C)** Tumor latency in SCID mice after an injection of 250,000 MCF7 or MCF7hPRL cells -/+ doxorubicin over 60 days. **(D)** Comparison of accumulated tumor volumes between treatment groups over 60 days. The sample size is *n* = 5 mice for each group. Log-rank (Mantel–Cox) and Gehan–Breslow Wilcoxon tests were used for the statistical analysis. Tumor volumes were tested using one-way ANOVA followed by Brown–Forsythe test.

To test the effect of autocrine PRL on the tumorigenicity, latency, and tumor size of breast cancer cells in the xenograft model, 500,000 MCF7hPRL or MCF7 control cells pre-treated with doxorubicin were injected into the mammary fat pad and monitored for 60 days. There was a longer latency to tumor formation for PRL-secreting, doxorubicin-treated MCF7hPRL cells compared to all other conditions, and it was significantly different than the MCF7hPRL tumors (log-rank *P* = .039, Gehan–Breslow Wilcoxon, *P* = .02) (**Figure 1A**). The tumor volume also appeared smaller with doxorubicin-treated MCF7hPRL cells (**Figure 1B**).

To be able to more subtly observe the latency time between the groups, we repeated the experiment with a reduced number of injected cells (250,000 cells). Consistent with the first experiment, increased latency was observed in the mice injected with doxorubicin-treated MCF7hPRL cells, as tumor formation was observed at 34 days after cell injection and detected only in 20% of mice over 60 days. This delay was statistically different when compared with the group of mice injected with MCF7hPRL cells (log-rank *P* = .042, Gehan–Breslow Wilcoxon *P* = .039) at 60 days (**Figure 1C**).

Consistent with the previous experiment, the tumors generated by doxorubicin-treated MCF7hPRL cells were very small in volume and statistically different when compared with the tumors formed with MCF7 control cells (*P*<.001) (**Figure 1D**). Upon allowing the tumors to grow over 120 days, all animals acquired a tumor, and the delay in latency observed in doxorubicin-treated MCF7hPRL cells was not permanent (**Supplementary Figure S2A**).

Overall, we observed that latency to tumor formation in SCID mice was significantly delayed in MCF7hPRL cells pre-treated with doxorubicin and resulted in the smallest tumors independent of cell number.

### 3.4 Autocrine PRL and DNA damage response attracts asialo-GM1-positive immune cells in SCID mice

To determine if the reason for the delayed latency to tumor formation in the MCF7hPRL cells with doxorubicin-induced DNA damage was due in part to the increased activity or the presence of immune cells, such as NK cells, we assessed the involvement of asialo-GM1-positive immune cells in the mechanism. We used the same recurrence animal model, injecting 500,000 MCF7 control or MCF7hPRL cells, treated or not with doxorubicin, in each group of SCID mice. Anti-asialo-GM1 antibody or control serum was injected intraperitoneally from the start of the experiment to address the effect on tumor initiation.

**Figure 2:**
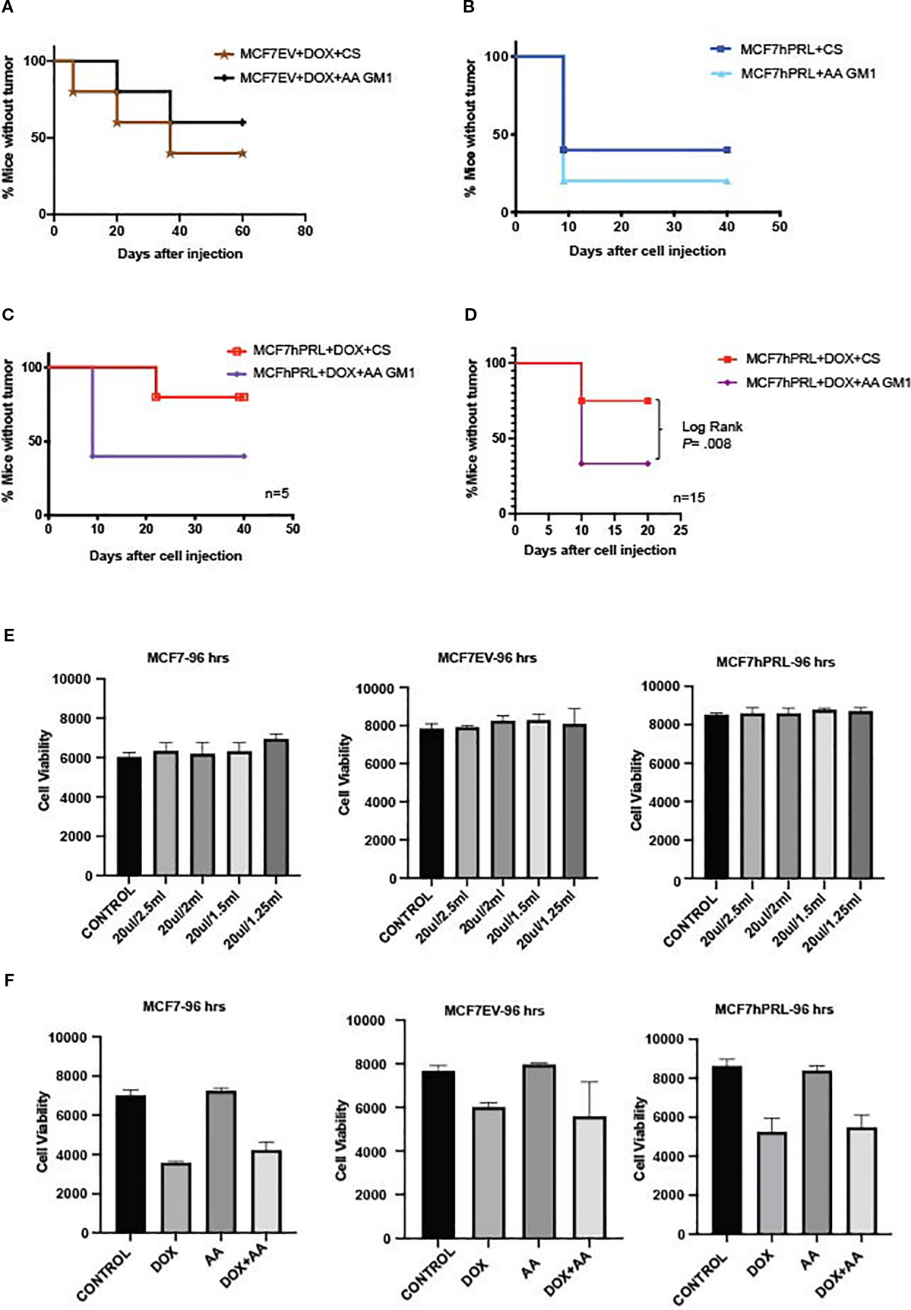
Autocrine PRL and DNA damage response in breast cancer cells attract anti-asialo GM1 immune cells in SCID mice. **(A)** Tumor latency after an injection of 500,000 doxorubicin-treated MCF7EV cells in mice treated with anti-asialo (AA) GM1 or control serum (CS). The sample size is *n* = 5 mice for each group. **(B)** Tumor latency after an injection of 500,000 MCF7hPRL cells in mice treated with anti-asialo GM1 or control serum. The sample size is *n* = 5 mice for each group. **(C)** Tumor latency after an injection of 500,000 doxorubicin-treated MCF7hPRL cells in mice treated with anti-asialo GM1 or control serum. The sample size is *n* = 5 mice for each group. Log-rank (Mantel–Cox) and Gehan–Breslow Wilcoxon tests were used for the statistical analysis. **(D)** Tumor latency after an injection of 10^6^ doxorubicin-treated MCF7hPRL cells in mice treated with anti-asialo GM1 or control serum. The sample size is *n* = 15 mice for each group. Log-rank (Mantel–Cox) and Gehan–Breslow Wilcoxon tests were used for the statistical analysis. **(E)** Cell viability (Alamar blue) assay showing that anti-asialo GM1 does not affect the viability of MCF7, MCF7EV, and MCF7hPRL cells. The cells were treated with 20 µL/2.5 mL, 20 µL/2 mL, 20 µL/1.5 mL, and 20 µL/1.25 mL of 1.1 mg of anti-asialo GM1. The cell viability was observed over 96 h Graphs represent pooled experiments, *n* = 6. **(F)** Cell viability (Alamar blue) assay showing anti-asialo GM1 does not affect the viability of MCF7, MCF7EV, and MCF7hPRL cells in the presence and absence of doxorubicin. The cells were treated with 20 µL/2.5 mL, 20 µL/2 mL, 20 µL/1.5 mL, and 20 µL/1.25 mL of anti-asialo GM1. The cell viability was followed for 96 h The graphs represent pooled experiments, *n* = 6.

Importantly, tumor initiation was independent of anti-asialo-GM1 injection in mice injected with doxorubicin-treated MCF7EV cells and untreated MCF7hPRL cells when compared to control serum treatment (**Figures 2A, B**). Tumor formation was, however, increased by anti-asialo GM1 injection in mice injected with doxorubicin-treated MCF7hPRL cells (**Figure 2C**). We confirmed our findings in a repeat experiment using 1 × 10^6^ MCF7hPRL cells treated with doxorubicin and 15 mice per group instead of five animals. The anti-asialo GM1 injection resulted in tumor formation in 75% of the mice, whereas only 25% of the mice formed tumor in the control group in 10 days (log-rank *P* = .008, Gehan–Breslow Wilcoxon *P* = .008) (**Figure 2D**). Therefore, combined PRL and doxorubicin treatments resulted in delayed tumor initiation due largely to the action of asialo-GM1-positive immune cells.

To rule out the effect of anti-asialo GM1 treatment on the viability of the breast cancer cells, we used an *in vitro* cell viability assay and treated breast cancer cells in the presence or absence of doxorubicin treatment. Anti-asialo GM1 did not have any effect on cell viability with or without doxorubicin at 24 - 72 h (**Supplementary Figure S3**) or with doxorubicin at 96 h (**Figures 2E, F**). These results confirmed that the observed effects of anti-asialo treatment in mice were due specifically to the impact on anti-asialo GM1-positive cells.

### 3.5 NK and macrophage recruitment changes after breast cancer cell and anti-asialo GM1 injections

Anti-asialo GM1 antibody was originally designed to deplete NK cells (27), but it can also deplete other immune cells, such as macrophages (28). Since DNA damage response attracts NK cells (29–31), we examined the injected mammary glands to understand NK and macrophage recruitment and depletion by the anti-asialo-GM1 antibody. To investigate the effect of DNA damaged breast cancer cells secreting autocrine PRL on immune cell trafficking, we injected 1 × 10^6^ doxorubicin-treated MCF7hPRL cells, and after 10 days, we examined the recruitment of NK cells and macrophages in both injected and contralateral mammary glands by FACS analysis using the following markers: CD45 as a nucleated hematopoietic marker for immune cells (32), DX5 (CD49b, Integrin alpha 2) as the marker for the general NK population in SCID mice, NKp46 as the major NK marker triggering antitumor activity and natural cytotoxicity (26, 33), and F4/80 as a macrophage cell marker (34).

**Figure 3:**
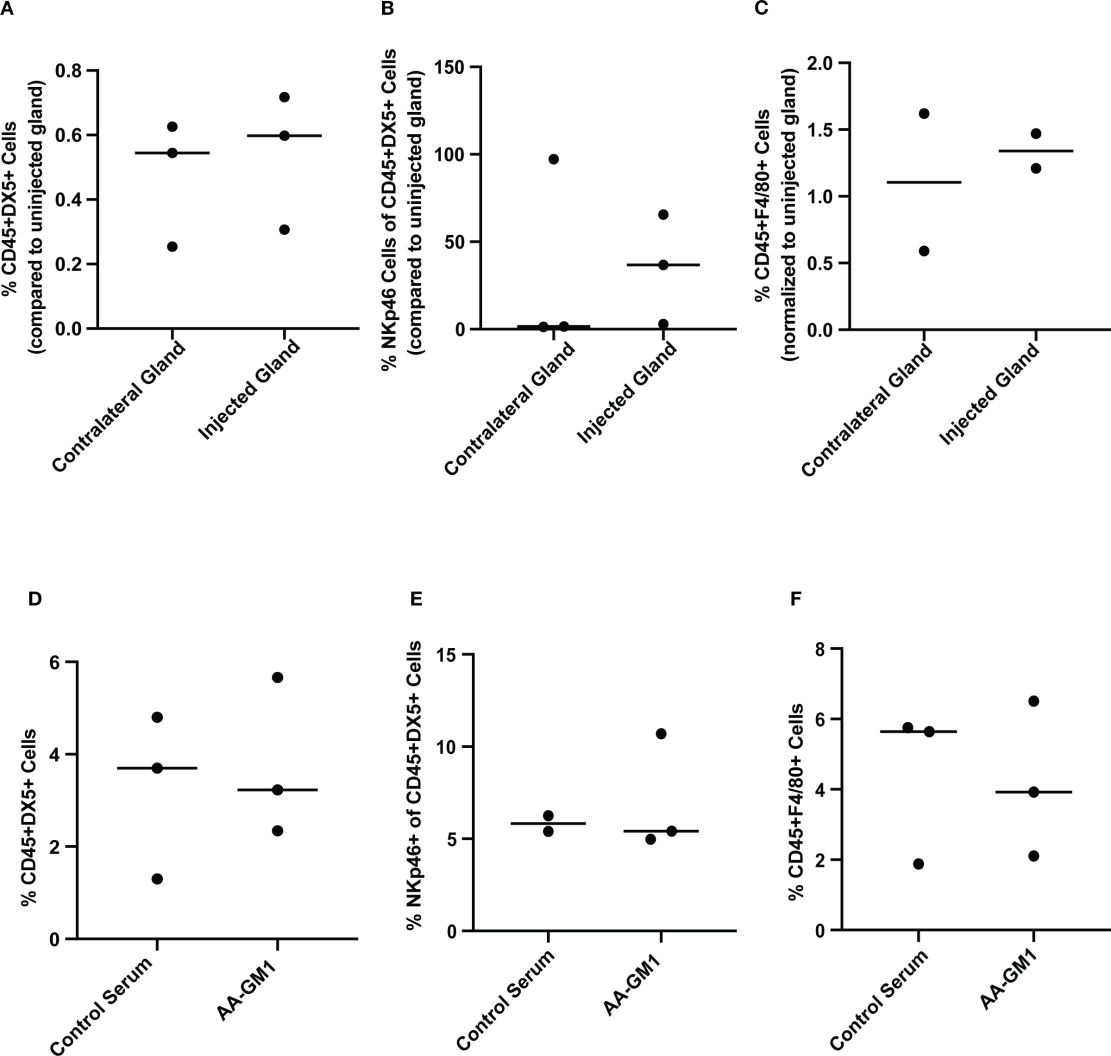
Percentage of NK cells and macrophages in SCID mice mammary gland after injecting DNA-damaged autocrine prolactin-secreting breast cancer cells. MCF7EV and MCF7hPRL cells were seeded and treated with 1 uM doxorubicin for 2 h on the following day. After 48 h of recovery time, 10^6^ cells were injected into SCID mice mammary glands. (**A**–**C**) The mammary glands were collected 10 days after cell injection. The injected and uninjected glands were processed separately (*n* = 3). The injected glands from *n* = 3 mice were pooled for staining (*n* = 2), and their contralateral glands were pooled for staining (*n* = 2) and uninjected mouse mammary gland (*n* = 3 mice), the glands were pooled for staining (*n* = 1). **(A)** Relative percentage of CD45+DX5+ cells. **(B)** Relative percentage of Nkp46+ cells within CD45+DX5+ cells. **(C)** %CD45+F4/80+ cells. For (**A**–**C**), in all cases, the cell numbers were normalized to those seen in uninjected cells. (**D**–**F**) The percentage of cells was evaluated from mammary glands after anti-asialo GM1 and control serum treatment after 25 days. For the anti-asialo GM1 group (*n* = 6 mice), the glands were pooled for staining (*n* = 3), and for the control serum group (*n* = 6 mice), the glands were pooled for staining (*n* = 3). **(D)** %CD45+DX5+cells. **(E)** %NKp46+ cells within CD45+DX5+ cells. **(F)** %CD45+F4/80+ cells.

The results showed an increasing trend in the recruitment of NK cells and macrophages, especially in the cytotoxic NKp46+ cells after 10 days (**Figures 3A–C**). The glands were also examined at the end of the anti-asialo GM1 injection experiment at day 25. The anti-asialo GM1 treatment caused a reduced trend in the macrophage cell population after 25 days of treatment compared to control serum injection (**Figures 3D–F**). We identified two NK populations and macrophage cells in the mammary glands.

### 3.6 DNA damage and PRL alter NK ligand levels in breast cancer cells

The major histocompatibility complex (MHC) known as human leukocyte antigen (HLA) class I molecules (HLA-A, HLA-B, and HLA-C) are primary regulators of NK cell activation (35). Upon cellular stress and DNA damage, HLA class 1 levels may be downregulated, and activating receptors such as natural killer group 2D (NKG2D) and DNAX accessory molecule 1 (DNAM-1) are upregulated (36) in an ATM- and ATR-dependent manner (37).

**Figure 4:**
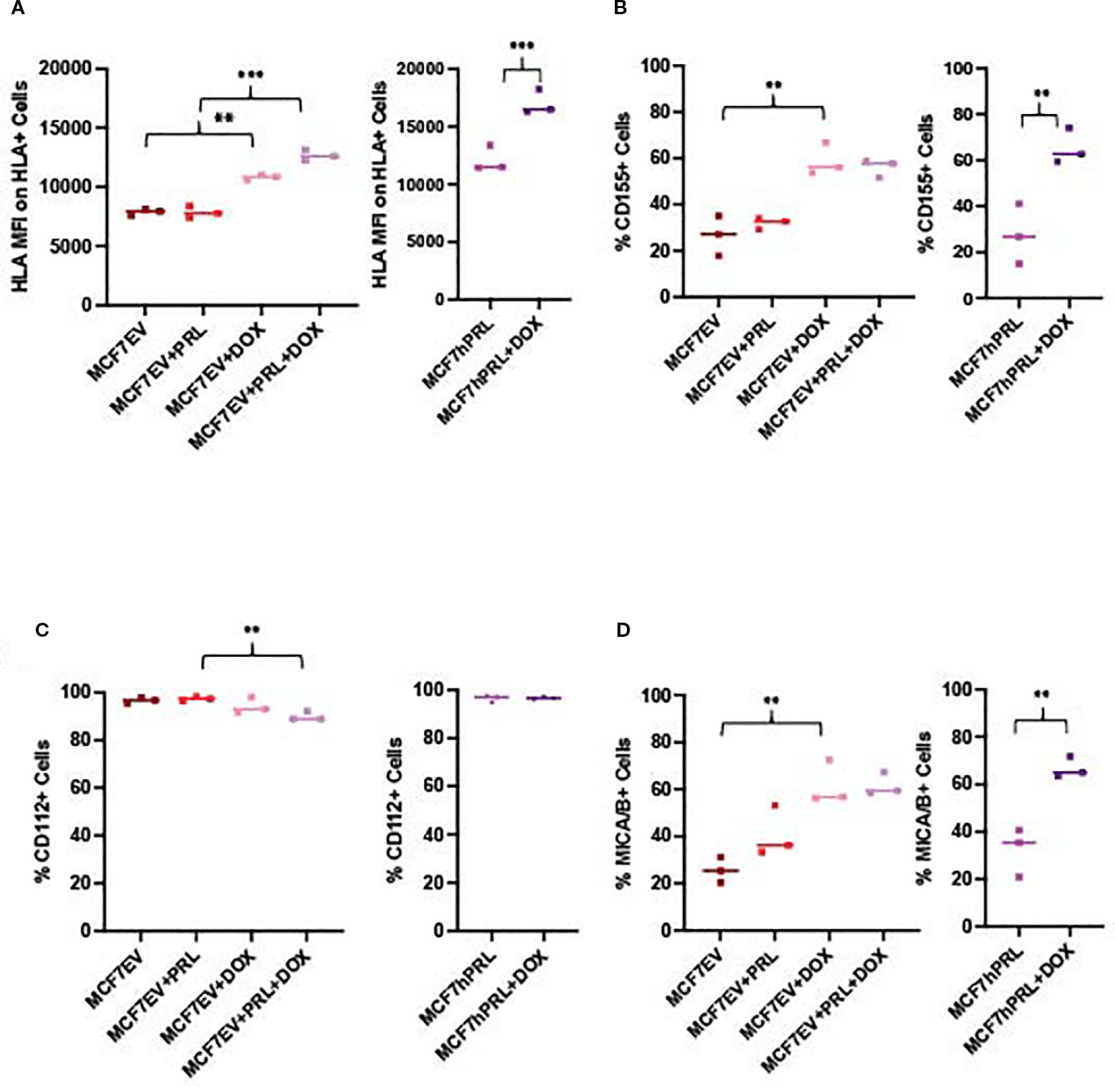
DNA damage and prolactin alters NK ligand expression in breast cancer cells. FACS analysis measuring the NK ligands from MCF7hPRL and MCF7EV cells in the presence or absence of doxorubicin treatment. Cells, 1 × 10^6^, were seeded, and MCF7EV cells were pre-treated with 25 ng/mL human recombinant prolactin for 24 h, followed by 2 h of doxorubicin treatment (1 uM). The cells were trypsinized after 48 h of recovery time and stained with CD155, CD112, MICA/B, and HLA markers for FACS analysis. The graphs represent a pooled experiment, *n* = 3. **(A)** HLA median fluorescence intensity on HLA+ MCF7EV cells and HLA+ MCF7hPRL cells. **(B)** %CD155+ MCF7EV and MCF7hPRL cells. **(C)** %MICA/B+ MCF7EV and MCF7hPRL cells. **(D)** %CD112+ MCF7EV and MCF7hPRL cells. Statistically significant analysis *P<.05, **P<.01, ***P<.001.

We evaluated HLA (HLA-A, HLA-B, and HLA-C) levels from MCF7EV and MCF7hPRL following doxorubicin and PRL treatment by using FACS analysis. We determined a significant increase in HLA median fluorescence intensity, particularly in doxorubicin-treated cells, in the presence of PRL. HLA staining was 1.6-fold higher in doxorubicin-treated MCF7EV cells compared to PRL-treated cells (*P*<. 001) and 1.4-fold higher in doxorubicin-treated MCF7hPRL cells compared to untreated cells (*P*<.001) (**Figure 4A**). These results demonstrated that HLA class I molecules are upregulated by the DNA damage response and higher in the presence of PRL.

To identify breast cancer ligands involved in triggering NK cell-activating receptors, we investigated the protein levels of CD155 and CD112 as DNAM-1 ligands (38) and MICA/B as NKG2D ligand (29) from MCF7EV and MCF7hPRL cells by using FACS analysis under the same experimental conditions. The %CD155 levels showed an increase in doxorubicin-treated cells when compared to the untreated controls; it was 2.1-fold higher in MCF7EV cells (*P* = .003) and 2.4-fold higher in MCF7hPRL cells (*P*<.001) (**Figure 4B**). The %CD112 levels demonstrated a decrease with PRL plus doxorubicin treatment compared to the PRL-treated (P = .003) MCF7EV cells (**Figure 4C**). The %MICA/B levels demonstrated a significant increase in doxorubicin-treated cells compared to the untreated controls; it was 2.4-fold higher in MCF7EV cells (*P* = .001) and twofold higher in MCF7hPRL cells (*P* = .002) (**Figure 4D**).

Overall, the results demonstrated that NK ligands CD155 and MICA/B are upregulated in the presence of PRL and DNA damage, likely driven by doxorubicin treatment. These results confirmed that some NK-activating ligands are upregulated in the presence of PRL and DNA damage response, likely mostly driven by DNA damage.

### 3.7 DNA damage response increases senescence

It is well known that DNA damage induces permanent cell cycle arrest, known as senescence. Cancer cells with WT p53, like MCF7 cells, undergo senescence in response to chemotherapy treatment (39). To investigate if senescence is part of the mechanism for increased latency and small tumor formation from doxorubicin-treated autocrine PRL-secreting cells, we measured the senescence-associated beta-galactosidase levels.

**Figure 5:**
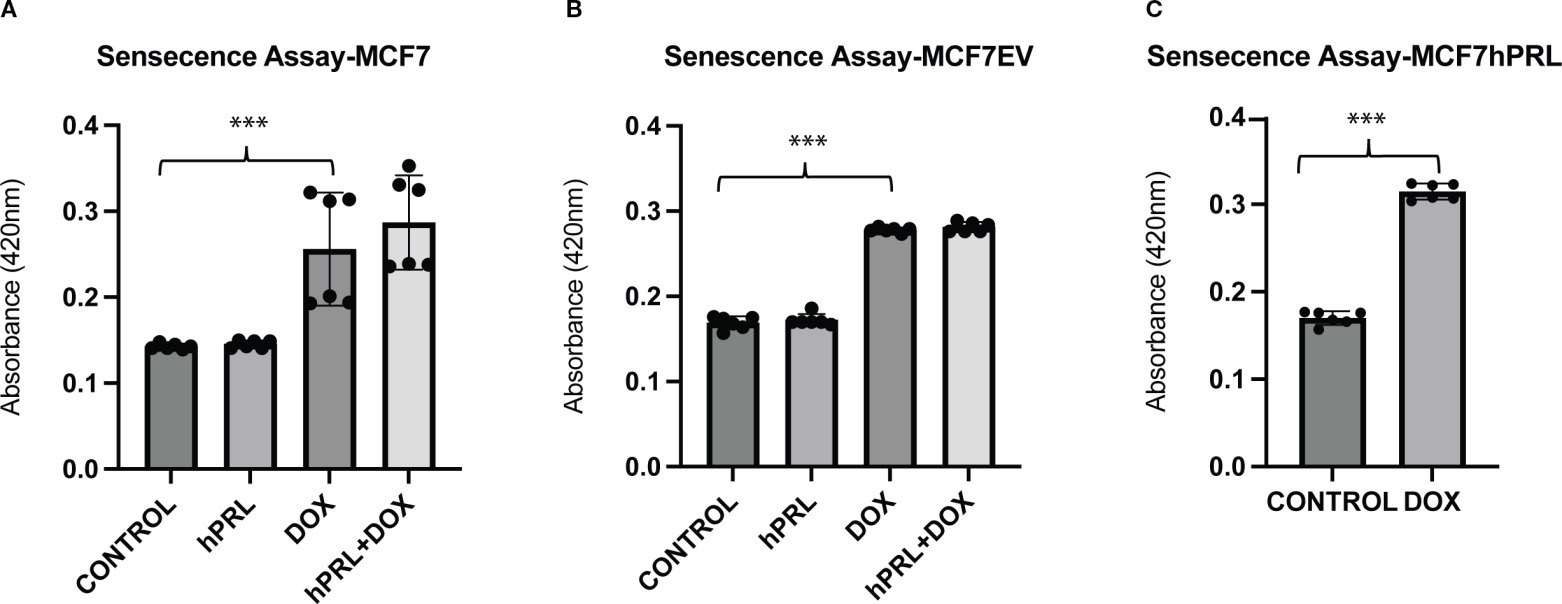
Autocrine prolactin and DNA damage increases cellular senescence (**A**–**C**). Determining the increased effect of doxorubicin on senescence in MCF7hPRL, MCF7EV, and MCF7 cells. The MCF7 and MCF7EV cells were pre-treated with human recombinant prolactin (25 ng/mL) for 24 h, followed by 2 h of doxorubicin treatment (1 µM). The cells were recovered in the presence or absence of prolactin for 6 days. The ONPG levels were measured by using a spectrophotometer. The graph represents six independent experiments. Statistically significant analysis ***P<.001.

As expected, doxorubicin treatment increased the senescence significantly in MCF7 and MCF7EV cell lines compared to the control cells (*P* = .008, *P*<.001). However, there was no significant difference detected with the addition of recombinant PRL (**Figures 5A, B**). Doxorubicin treatment significantly increased the senescence in MCF7hPRL cells compared to the control group (*P*<.001) (**Figure 5C**). This result confirmed that breast cancer cells go under DNA-damage-induced senescence likely independent of PRL.

### 3.8 Autocrine PRL and DNA damage response increase NK cell-mediated killing

**Figure 6:**
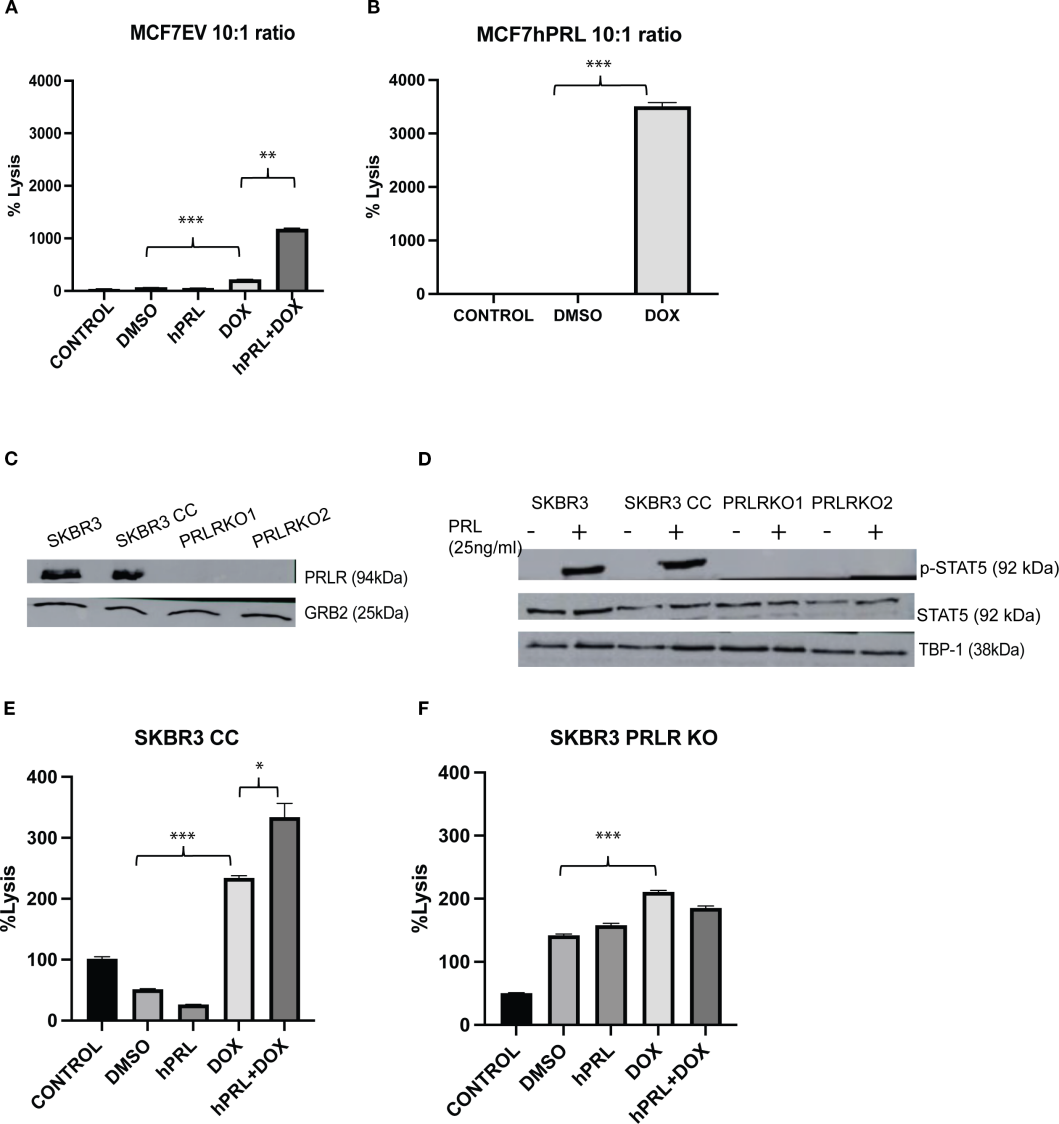
Autocrine prolactin and DNA damage increases NK cell-mediated cell lysis *in vitro*. **(A, B)** Calcein-AM assay determining the NK cell-mediated lysis of MCF7hPRL and MC7EV cells in the presence or absence of DNA damage. The MCF7EV cells were pre-treated with human recombinant prolactin (25 ng/mL) for 24 h, followed by 2 h of doxorubicin treatment (1 uM). The cells were trypsinized after 48 h of recovery time and co-cultured with NK cells in a 10:1 effector/target ratio. The cell viability of breast cancer cells was determined by Calcein-AM assay and the %lysis was calculated. **(C)** PRLR and GRB2 (loading control) protein levels from SKBR3, SKBR3 CC, SKBR3PRLRKO1, and SKBR3PRLRKO2 cells. **(D)** p-STAT5 and total STAT5 and TBP-1 (loading control) protein levels from SKBR3, SKBR3 CC, SKBR3PRLRKO1, and SKBR3PRLRKO2 cells treated or not with 25 ng/mL human recombinant PRL. **(E)** Calcein-AM assay determining the NK cell-mediated lysis of SKBR3CC (Crispr control cells) cells in the presence or absence of DNA damage at 1:10 effector/target ratio. **(F)** Calcein-AM assay determining the NK cell-mediated lysis of a PRLR knockout cell clone (PRLRKO1) in the presence or absence of DNA damage at 1:10 effector/target ratio. Statistically significant analysis *P<.05, **P<.01, ***P<.001.

Given that the anti-asialo GM1 antibody is well known to deplete NK cells and that we observed NK cells in the tumors, we investigated whether NK cell cytotoxicity may contribute to our observation of delayed latency after autocrine PRL and doxorubicin treatment. DNA damage has been demonstrated to activate several ligands specific for NK cell receptors (reviewed in (36)). Chemotherapeutic agent-induced cellular stress and DNA damage response do attract NK cells (29–31). To examine if the combination of autocrine PRL secretion and DNA damage response makes breast cancer cells more susceptible to NK cells, we performed a Calcein-AM assay to evaluate the percent lysis of breast cancer cells by NK cells under different experimental conditions. Human NK92MI NK cells were co-cultured with doxorubicin-treated and untreated MCF7, MCF7EV, and MCF7hPRL at 10:1 and 1:1 effector/target ratios. To compare the effect of autocrine and recombinant PRL, the MCF7 and MCF7EV cells were also treated with 25 ng/mL human recombinant PRL in the indicated groups. The results demonstrated that DNA damage increases NK-mediated lysis significantly in all three MCF7 lines at 1:1 (**Supplementary Figure S4**) and 10:1 ratios (**Supplementary Figure S4D**, **Figure 6A, B**). In the MCF7EV cells, human recombinant PRL and doxorubicin treatment increased the percentage of lysis fivefold compared to doxorubicin alone (*P* = .001) (**Figure 6A**). The strongest effect was observed in autocrine PRL-secreting cells treated with doxorubicin, with the percentage of lysis 133-fold higher compared to the untreated control (*P*<.001) and 220-fold higher compared to the vehicle-treated control (*P*<.001) (**Figure 6B**). The results confirmed that doxorubicin and PRL, particularly autocrine PRL, increases the susceptibility of breast cancer cells to the cytotoxicity of NK cells.

### 3.9 Active PRLR and DNA damage response increase NK cell-mediated killing

To further evaluate if the NK killing is due to the PRLR on breast cancer cells, we performed Calcein-AM assays with SKBR3 cells that have a CRISPR/Cas9-mediated deletion of the PRLR gene [SKBR3PRLR-knockout-(KO)] and CRISPR Control (CC) SKBR3 cells. We validated the PRLR KO with western blots (**Figure 6C**). As expected, there was no STAT5 phosphorylation in the PRLR KO lines compared to the SKBR3 and SKBR3-CC cells treated with 25 ng/mL human recombinant PRL (**Figure 6D**).

We performed Calcein-AM assays in co-cultures with NK92MI cells and SKBR3 cell lines at a 10:1 effector/target ratio (**Supplementary Figure S4E**, **Figure 6E, F**). In SKBR3 CC cells, doxorubicin treatment increased the NK-mediated lysis significantly compared to the vehicle control (*P*<.001), and PRL plus doxorubicin increased lysis significantly compared to PRL (*P* = .005) or doxorubicin alone (*P* = .01) or (**Figure 6E**). In SKBR3PRLR-KOs, although doxorubicin induced lysis by NK cells (*P*<.001), there was no PRL-mediated increase in lysis in the doxorubicin-treated cells, indicating that the effect of PRL on NK lysis was PRLR-mediated (**Figure 6F**). Therefore, we confirmed increased NK-mediated lysis with doxorubicin treatment in SKBR3, SKBR3 CC, and SKBR3PRLR-KO cells. PRL plus doxorubicin treatment resulted in a further increase in NK-mediated lysis in SKBR3CC cells, and this was abrogated by the loss of the PRLR.

## 4 Discussion

In our breast cancer recurrence model, we demonstrated that autocrine PRL in the tumor microenvironment does, in fact, not delay tumor initiation, but in the context of the DNA damage response (15), combined PRL signaling with DNA damage leads to immune cell attack, in particular by cytotoxic NK cells, and increases the latency of tumor formation. These observations may clarify the role of PRL and the PRLR in breast cancer tumorigenesis and tumor progression.

In our previous study (15), we demonstrated that PRL increases cellular viability against DNA damaging chemotherapy agents and established a PRLR-dependent crosstalk between the PRL-Stat5 pathway and the ATM DNA damage repair pathway. It is well known that the ATM-mediated DNA damage response alters breast cancer NK ligand levels and attracts more NK cells (36). We suggest that the crosstalk between PRL/PRLR signaling and DNA repair pathways affects breast cancer NK cell activating ligand levels as well as cytokine/chemokine secretion, making breast cancer cells more susceptible to NK cell-mediated killing. On the other hand, NK cells are known to have PRLR and their activation is dependent on PRLR and PRL (40). We believe that the autocrine PRL that is secreted from breast cancer cells may also affect their activation and attraction in the breast cancer microenvironment.

Previous studies indicated the potential involvement of PRL and PRLR in immune and inflammatory responses in breast cancer (41, 42). In breast cancer patients, the differentially released immunosuppressive cytokine interleukin (IL)-10 altered PRLR expression in peripheral blood mononuclear cells and reduced the PRL-mediated anti-inflammatory response (42). In a recent study, knockdown of long-form PRLR in metastatic triple-negative breast cancer cells reduced metastasis and increased survival, reducing T regulatory cell recruitment to the tumor by modulating the CCL17 levels (41). While the breast cancer subtypes differ in this study compared to ours, it is evident that the prolactin receptor can modulate the adaptive immune response. These previous studies, however, did not address the recruitment of other immune cell types, such as NK cells, to tumor or its microenvironment.

NK cells are an important factor in cancer cell dormancy (43, 44), and cancer cell dormancy is an important clinical consideration, in particular for estrogen receptor-positive breast cancer patients (45). Our research has shown that high levels of the PRLR in the primary breast tumor shortens the time to bone metastasis in human patients (4). Recently, a fusion protein was created, consisting of the extra-cellular portion of MICA and a variant of PRL, G129R, that activates NK cells specifically on PRLR-positive cells as a new approach for breast-cancer-specific immunotherapy (46).

The anti-asialo antibody was designed to deplete NK cells, but previous studies have discovered that it can also target basophil cells (47), NK, NKT, CD8+T, γδT, some CD4+T cells, macrophages, and eosinophils (28, 47–50). Although SCID mice do not have functional B or T cells, they do have functional NK cells, macrophages, eosinophils, and basophils (18), of which NK cells (51), eosinophils (52), basophils (53), and macrophages (54) have been implicated in anti-tumor activity.

PRL has been previously implicated in the recruitment of immune cells to the mammary gland though it was not until that it was reported that autocrine PRL recruited NK cells to breast cancer cells. PRL treatment of female mice increased the lymphocytes in the mammary gland (55). PRL treatment of mammary HC11 cells also increased the migration of B cells, CD4+ T cells, CD4+ memory T cells, CD8+ memory cells, macrophages, monocytes, neutrophils, eosinophils (55), and basophils (47, 56). PRL was also shown to directly improve the antitumor effects of NK cells in Balb/c and SCID mice via the PRLR on NK cells (40, 57, 58). PRL has been shown to directly upregulate the NK major receptors involved in lytic cell death (59). In our study, we found that breast cancer cells have increased levels of certain ligands, known to activate major NK receptors, in the presence of PRL and DNA damage response. This could be part of the mechanism of the observed antitumor effect in SCID mice.

In summary, we observed that PRL supports mammary tumor formation, but that PRL in combination with the DNA damage response increases the tumor latency of breast cancer cells in an orthotopic xenograft model in a mechanism that involves asialo-GM1-expressing immune cells, likely NK cells. This may have implications for the use of PRLR antagonists during anti-cancer treatment with doxorubicin.

## Data Availability

The original contributions presented in the study are included in the article/material. Further inquiries can be directed to the corresponding author.

## References

[1] Shemanko CS. Prolactin receptor in breast cancer: marker for metastatic risk. J Mol Endocrinol. (2016) 57:R153–R65. doi: 10.1530/JME-16-0150, PMID: 27658959

[2] Horseman ND Zhao W Montecino-Rodriguez E Tanaka M Nakashima K Engie SJ. Defective mammopoiesis, but normal hematopoiesis, in mice with a targeted disruption of the prolactin gene. EMBO J. (1997) 16:6926–35. doi: 10.1093/emboj/16.23.6926, PMID: 9384572 PMC1170296

[3] Arendt LM Schuler LA. Transgenic models to study actions of prolactin in mammary neoplasia. J mammary gland Biol neoplasia. (2008) 13:29–40. doi: 10.1007/s10911-008-9073-9, PMID: 18219562

[4] Sutherland A Forsyth A Cong Y Grant L Juan TH Lee JK. The role of prolactin in bone metastasis and breast cancer cell-mediated osteoclast differentiation. J Natl Cancer Institute. (2016) 108:djv338. doi: 10.1093/jnci/djv338, PMID: 26586670 PMC5943829

[5] Yonezawa T Chen KH Ghosh MK Rivera L Dill R Ma L. Anti-metastatic outcome of isoform-specific prolactin receptor targeting in breast cancer. Cancer letters. (2015) 366:84–92. doi: 10.1016/j.canlet.2015.06.010, PMID: 26095602

[6] Ben-Jonathan N Mershon JL Allen DL Steinmetz RW. Extrapituitary prolactin: distribution, regulation, functions, and clinical aspects. Endocr Rev. (1996) 17:639–69. doi: 10.1210/edrv-17-6-639, PMID: 8969972

[7] Muthuswamy SK. Autocrine prolactin: an emerging market for homegrown (prolactin) despite the imports. Genes Dev. (2012) 26:2253–8. doi: 10.1101/gad.204636.112, PMID: 23070811 PMC3475797

[8] Arendt LM Rugowski DE Grafwallner-Huseth TA Garcia-Barchino MJ Rui H Schuler LA. Prolactin-induced mouse mammary carcinomas model estrogen resistant luminal breast cancer. Breast Cancer Res. (2011) 13:R11. doi: 10.1186/bcr2819bcr2819, PMID: 21276249 PMC3109579

[9] Liby K Neltner B Mohamet L Menchen L Ben-Jonathan N. Prolactin overexpression by MDA-MB-435 human breast cancer cells accelerates tumor growth. Breast Cancer Res Treat. (2003) 79:241–52. doi: 10.1023/A:1023956223037, PMID: 12825859

[10] Rose-Hellekant TA Arendt LM Schroeder MD Gilchrist K Sandgren EP Schuler LA. Prolactin induces ERalpha-positive and ERalpha-negative mammary cancer in transgenic mice. Oncogene. (2003) 22:4664–74. doi: 10.1038/sj.onc.1206619, PMID: 12879011 PMC1630768

[11] Sun Y Yang N Utama FE Udhane SS Zhang J Peck AR. NSG-Pro mouse model for uncovering resistance mechanisms and unique vulnerabilities in human luminal breast cancers. Sci Adv. (2021) 7:eabc8145. doi: 10.1126/sciadv.abc8145, PMID: 34524841 PMC8443188

[12] Wennbo H Gebre-Medhin M Gritli-Linde A Ohlsson C Isaksson OG Tornell J. Activation of the prolactin receptor but not the growth hormone receptor is important for induction of mammary tumors in transgenic mice. J Clin Invest. (1997) 100:2744–51. doi: 10.1172/JCI119820, PMID: 9389738 PMC508478

[13] Lopez-Ozuna VM Hachim IY Hachim MY Lebrun JJ Ali S. Prolactin modulates TNBC aggresive phenotype limiting tumorigenesis endocrine-related cancer. Endocr Relat Cancer. (2019) 26:321–37. doi: 10.1530/ERC-18-0523, PMID: 30640712

[14] Grible JM Zot P Olex AL Hedrick SE Harrell JC Woock AE. The human intermediate prolactin receptor is a mammary proto-oncogene. NPJ Breast Cancer. (2021) 7:37. doi: 10.1038/s41523-021-00243-7, PMID: 33772010 PMC7997966

[15] Karayazi Atici O Urbanska A Gopinathan SG Boutillon F Goffin V Shemanko CS. ATM is required for the prolactin-induced HSP90-mediated increase in cellular viability and clonogenic growth after DNA damage. Endocrinology. (2018) 159:907–30. doi: 10.1210/en.2017-00652, PMID: 29186352

[16] LaPensee EW Ben-Jonathan N. Novel roles of prolactin and estrogens in breast cancer: resistance to chemotherapy. Endocrine-related cancer. (2010) 17:R91–107. doi: 10.1677/ERC-09-0253, PMID: 20071456

[17] Dewan MZ Terunuma H Ahmed S Ohba K Takada M Tanaka Y. Natural killer cells in breast cancer cell growth and metastasis in SCID mice. BioMed Pharmacother. (2005) 59 Suppl 2:S375–9. doi: 10.1016/s0753-3322(05)80082-4, PMID: 16507413

[18] Dorshkind K Pollack SB Bosma MJ Philips RA. Natural Killer (NK) cells are present in mice with severe combined immunodeficiency (scid). J Immunol. (1985) 134:3798–801. doi: 10.4049/jimmunol.134.6.3798, PMID: 3989296

[19] Kasai M Yoneda T Habu S Maruyama Y Okumura K Tokunaga T. *In vivo* effect of anti-asialo GM1 antibody on natural killer activity. Nature. (1981) 291:334–5. doi: 10.1038/291334a0, PMID: 7231554

[20] Perotti C Karayazi O Moffat S Shemanko CS. The bone morphogenetic protein receptor-1A pathway is required for lactogenic differentiation of mammary epithelial cells *in vitro* . In Vitro Cell Dev Biol Anim. (2012) 48:377–84. doi: 10.1007/s11626-012-9522-z, PMID: 22729646 PMC3404688

[21] Gary RK Kindell SM. Quantitative assay of senescence-associated beta-galactosidase activity in mammalian cell extracts. Anal Biochem. (2005) 343:329–34. doi: 10.1016/j.ab.2005.06.003, PMID: 16004951

[22] Somanchi SS McCulley KJ Somanchi A Chan LL Lee DA. A novel method for assessment of natural killer cell cytotoxicity using image cytometry. PloS One. (2015) 10:e0141074. doi: 10.1371/journal.pone.0141074, PMID: 26492577 PMC4619620

[23] Chatzistamou I Kiaris H. Modeling estrogen receptor-positive breast cancers in mice: is it the best we can do? Endocrine-related Cancer. (2016) 23:C9–C12. doi: 10.1530/ERC-16-0397, PMID: 27619257 PMC5063077

[24] Ingberg E Theodorsson A Theodorsson E Strom JO. Methods for long-term 17beta-estradiol administration to mice. Gen Comp Endocrinol. (2012) 175:188–93. doi: 10.1016/j.ygcen.2011.11.014, PMID: 22137913

[25] Krneta T Gillgrass A Chew M Ashkar AA. The breast tumor microenvironment alters the phenotype and function of natural killer cells. Cell Mol Immunol. (2016) 13:628–39. doi: 10.1038/cmi.2015.42, PMID: 26277898 PMC5037278

[26] Miao M Masengere H Yu G Shan F. Reevaluation of NOD/SCID mice as NK cell-deficient models. BioMed Res Int. (2021) 2021:8851986. doi: 10.1155/2021/8851986, PMID: 34805408 PMC8598338

[27] Yoshino H Ueda T Kawahata M Kobayashi K Ebihara Y Manabe A. Natural killer cell depletion by anti-asialo GM1 antiserum treatment enhances human hematopoietic stem cell engraftment in NOD/Shi-scid mice. Bone Marrow Transplant. (2000) 26:1211–6. doi: 10.1038/sj.bmt.1702702, PMID: 11149733

[28] Wiltrout RH Santoni A Peterson ES Knott DC Overton WR Herberman RB. Reactivity of anti-asialo GM1 serum with tumouricidal and non-tumouricidal mouse macrophages. J leukocyte Biol. (1985) 37:597–614. doi: 10.1002/jlb.37.5.597, PMID: 3884721

[29] Raulet DH Gasser S Gowen BG Deng W Jung H. Regulation of ligands for the NKG2D activating receptor. Annu Rev Immunol. (2013) 31:413–41. doi: 10.1146/annurev-immunol-032712-095951, PMID: 23298206 PMC4244079

[30] Raulet DH Guerra N. Oncogenic stress sensed by the immune system: role of natural killer cell receptors. Nat Rev Immunol. (2009) 9:568–80. doi: 10.1038/nri2604, PMID: 19629084 PMC3017432

[31] Soriani A Iannitto ML Ricci B Fionda C Malgarini G Morrone S. Reactive oxygen species- and DNA damage response-dependent NK cell activating ligand upregulation occurs at transcriptional levels and requires the transcriptional factor E2F1. J Immunol. (2014) 193:950–60. doi: 10.4049/jimmunol.1400271, PMID: 24913980

[32] Hermiston ML Xu Z Weiss A. CD45: a critical regulator of signaling thresholds in immune cells. Annu Rev Immunol. (2003) 21:107–37. doi: 10.1146/annurev.immunol.21.120601.140946, PMID: 12414720

[33] Abel AM Yang C Thakar MS Malarkannan S. Natural killer cells: development, maturation, and clinical utilization. Front Immunol. (2018) 9:1869. doi: 10.3389/fimmu.2018.01869, PMID: 30150991 PMC6099181

[34] Cansever D Petrova E Krishnarajah S Mussak C Welsh CA Mildenberger W. Lactation-associated macrophages exist in murine mammary tissue and human milk. Nat Immunol. (2023) 24:1098–109. doi: 10.1038/s41590-023-01530-0, PMID: 37337103 PMC10307629

[35] Cruz-Tapias P Castiblanco J Anaya JM. Major histocompatibility complex: Antigen processing and presentation. In: Anaya JM Schoenfeld Y Rojas-Villarraga A Levy RA Cervera R. editors. Autoimmunity: from bench to bedside. El Rosario University Press, Bogota, Colombia (2013). 29087650

[36] Chan CJ Smyth MJ Martinet L. Molecular mechanisms of natural killer cell activation in response to cellular stress. Cell Death differentiation. (2014) 21:5–14. doi: 10.1038/cdd.2013.26, PMID: 23579243 PMC3857624

[37] Soriani A Zingoni A Cerboni C Iannitto ML Ricclardi MR Di Gialleonardo V. ATM-ATR-dependent up-regulation of DNAM-1 and NKG2D ligands on multiple myloma cells by therapeutic agents results in enhanced NK-cell susceptibility and is associated with a senescent phenotype. Blood. (2009) 113:3503–11. doi: 10.1182/blood-2008-08-173914, PMID: 19098271

[38] Bottino C Castriconi R Pende D Rivera P Nanni M Carnemolla B. Identification of PVR (CD155) and Nectin-2 (CD112) as cell surface ligands for the human DNAM-1 (CD226) activating molecule. J Exp Med. (2003) 198:557–67. doi: 10.1084/jem.20030788, PMID: 12913096 PMC2194180

[39] George N Joshi MB Satyamoorthy K. DNA damage-induced senescence is associated with metabolomic reprogramming in breast cancer cells. Biochimie. (2024) 216:71–82. doi: 10.1016/j.biochi.2023.09.021, PMID: 37758157

[40] Sun RLAL Wei HM Tian ZG. Expression of prolactin receptor and response to prolactin stimulation of human NK cells. Cell Research. (2004) 14:67–73. doi: 10.1038/sj.cr.7290204, PMID: 15040892

[41] Chen KE Ghosh M Rivera L Lin S Kumar A Swaminathan S. Prolactin enhances T regulatory cell promotion of breast cancer through the long form prolactin receptor. Transl Oncol. (2021) 14:101195. doi: 10.1016/j.tranon.2021.101195, PMID: 34375938 PMC8358703

[42] Paul S Biswas A Sasmal K Mukherjee S Biswas T Biswas R. IL-10 alters prolactin receptor activity emulating that during breast cancer. Cytokine. (2010) 51:144–50. doi: 10.1016/j.cyto.2010.04.012, PMID: 20488724

[43] Bushnell GG Sharma D Wilmot HC Zheng M Fashina TD Hutchens CM. Natural killer cell regulation of breast cancer stem cells mediates metastatic dormancy. Cancer Res. (2024) 84:3337–53. doi: 10.1158/0008-5472.CAN-24-0030, PMID: 39106452 PMC11474167

[44] Correia AL Guimaraes JC Auf der Maur P De Silva D Trefny MP Okamoto R. Hepatic stellate cells suppress NK cell-sustained breast cancer dormancy. Nature. (2021) 594:566–71. doi: 10.1038/s41586-021-03614-z, PMID: 34079127

[45] Pan H Gray R Braybrooke J Davies C Taylor C McGale P. 20-Year Risks of Breast-Cancer Recurrence after Stopping Endocrine Therapy at 5 Years. N Engl J Med. (2017) 377:1836–46. doi: 10.1056/NEJMoa1701830, PMID: 29117498 PMC5734609

[46] Ding H Buzzard GW Huang S Sehorn MG Marcus RK Wei Y. MICA-G129R: A bifunctional fusion protein increases PRLR-positive breast cancer cell death in co-culture with natural killer cells. PloS One. (2021) 16:e0252662. doi: 10.1371/journal.pone.0252662, PMID: 34077462 PMC8172023

[47] Nishikado H Mukai K Kawano Y Minegishi Y Karasuyama H. NK cell-depleting anti-asialo GM1 antibody exhibits a lethal off-target effect on basophils *in vivo* . J Immunol. (2011) 186:5766–71. doi: 10.4049/jimmunol.1100370, PMID: 21490162

[48] Kataoka S Konishi Y Nishio Y Fujikawa-Adachi K Tominaga A. Antitumor activity of eosinophils activated by IL-5 and eotaxin against hepatocellular carcinoma. DNA and Cell Biology. (2004) 23:549–60. doi: 10.1089/dna.2004.23.549, PMID: 15383175

[49] Slifka MK Pagarigan RR Whitton JL. NK markers are expressed on a high percentage of virus-specific CD8+ and CD4+ T cells. J Immunol. (2000) 164:2009–15. doi: 10.4049/jimmunol.164.4.2009, PMID: 10657652

[50] Trambley J Bingaman AW Lin A Elwood ET Waitze SY Ha J. Asialo GM1(+) CD8(+) T cells play a critical role in costimulation blockade-resistant allograft rejection. J Clin Invest. (1999) 104:1715–22. doi: 10.1172/JCI8082, PMID: 10606625 PMC409885

[51] Yang Q Goding SR Hoklan ME Basse PH. Antitumor activity of NK cells. Immunologic Res. (2006) 36/1:13–25. doi: 10.1385/IR:36:1:13, PMID: 17337762

[52] Tepper RI. The eosinophil-mediated antitumor activity of interleukin-4. J Allergy Clin Immunol. (1994) 94:1225–30. doi: 10.1016/0091-6749(94)90336-0, PMID: 7798564

[53] Sektioglu IM Carretero R Bulbuc N Bald T Tuting T Rudensky AY. Basophils promote tumor rejection via chemotaxis and infiltration of CD8+ T cells. Cancer Res. (2017) 77:291–302. doi: 10.1158/0008-5472.CAN-16-0993, PMID: 27879269

[54] Komohara Y Kurotaki D Tsukamoto H Miyasato Y Yano H Pan C. Involvement of protumor macrophages in breast cancer progression and characterization of macrophage phenotypes. Cancer Sci. (2023) 114:2220–9. doi: 10.1111/cas.15751, PMID: 36748310 PMC10236637

[55] Dill R Walker AM. Role of prolactin in promotion of immune cell migration into the mammary gland. J mammary gland Biol neoplasia. (2017) 22:13–26. doi: 10.1007/s10911-016-9369-0, PMID: 27900586 PMC5313375

[56] Freeman ME Kanyicska B Lerant A Nagy G. Prolactin: structure, function, and regulation of secretion. Physiol Rev. (2000) 80:1523–631. doi: 10.1152/physrev.2000.80.4.1523, PMID: 11015620

[57] Sun RWH Zhang J Li A Zhang W Tian ZG. Recombinant human prolactin improves antitumor effecst of murine natural killer cells *in vitro* and *in vivo* . Neuroimmunomodulation (2002) 10:169–76. doi: 10.1159/000067179, PMID: 12481157

[58] Zhang J Sun R Wei H Tian Z. Antitumor effects of recombinant human prolactin in human adenocarcinoma-bearing SCID mice with human NK cell xenograft. Int Immunopharmacol. (2005) 5:417–25. doi: 10.1016/j.intimp.2004.10.008, PMID: 15652770

[59] Mavoungou E Bouyou-Akotet MK Kremsner PG. Effects of prolactin and cortisol on natural killer (NK) cell surface expression and function of human natural cytotoxicity receptors (NKp46, NKp44 and NKp30). Clin Exp Immunol. (2005) 139:287–96. doi: 10.1111/j.1365-2249.2004.02686.x, PMID: 15654827 PMC1809301

