# Supplementary figures and images for: Prolactin and DNA damage trigger an anti-breast cancer cell immune response
Source: Front Endocrinol (Lausanne). 2025 Sep 23;16:1586062. doi: 10.3389/fendo.2025.1586062 (PMC12500456; doi:10.3389/fendo.2025.1586062)

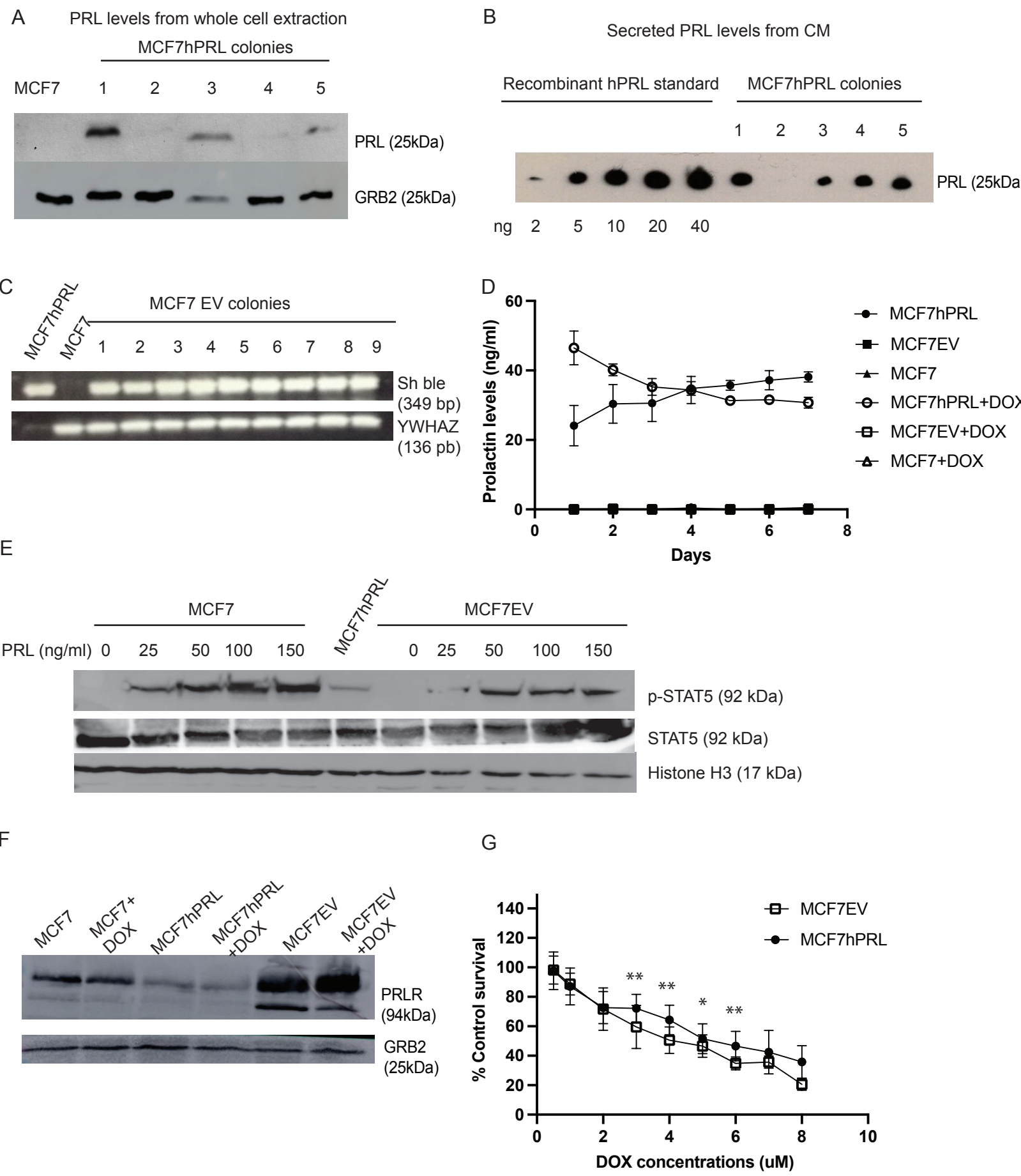

Supplement: Supplementary file 1 [file DataSheet1.pdf]

A

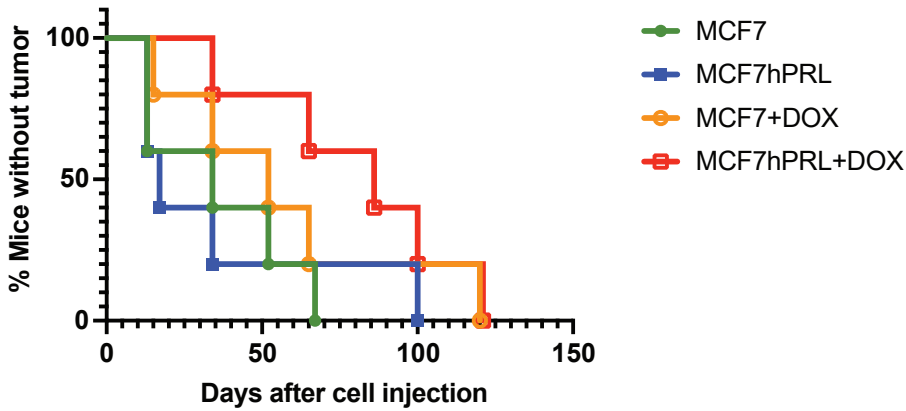

Supplement: Supplementary file 2 [file DataSheet2.pdf]

A

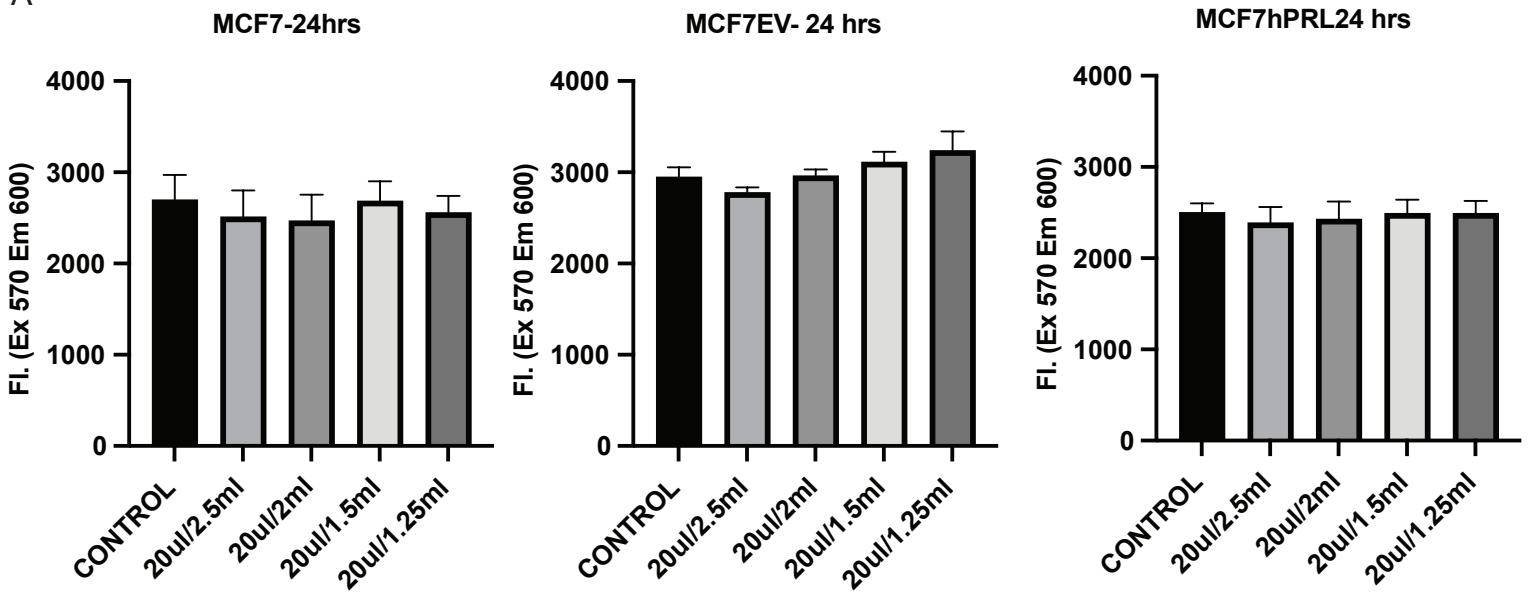

B

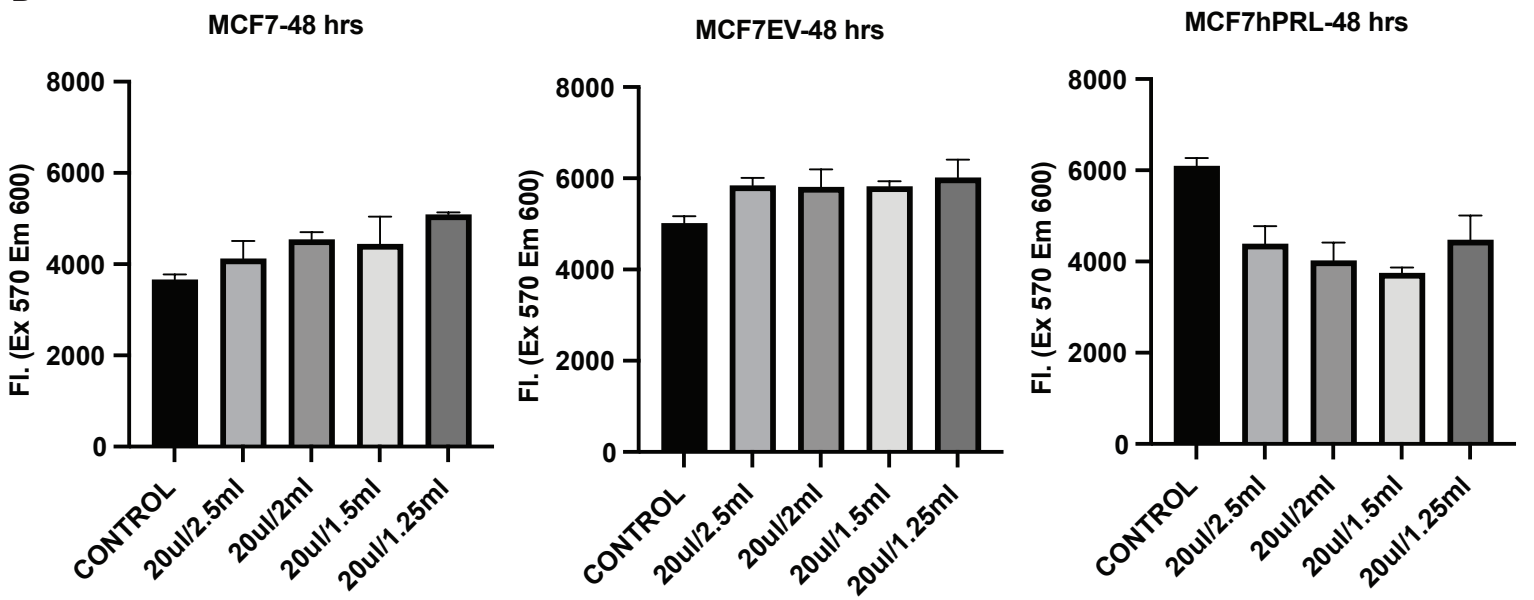

C

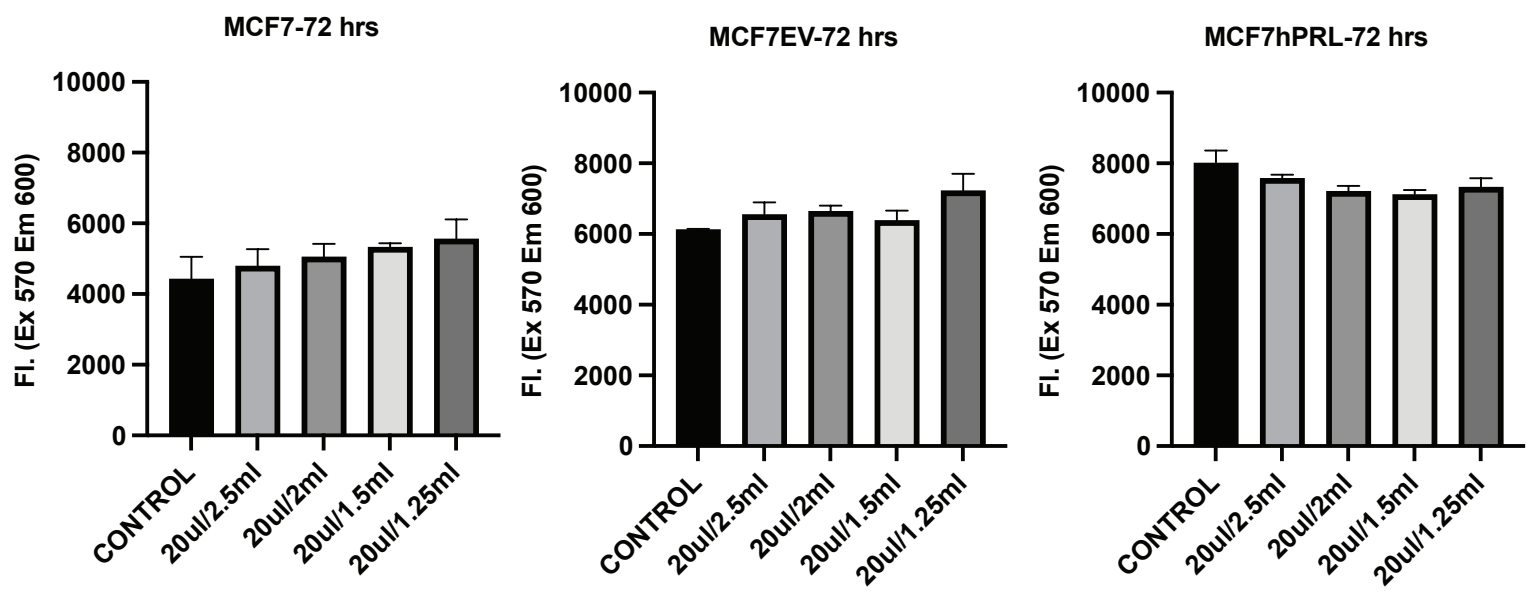

Supplement: Supplementary file 3 [file DataSheet3.pdf]

D

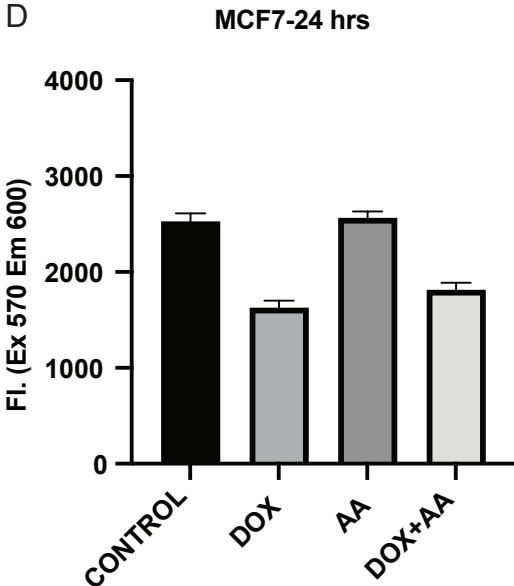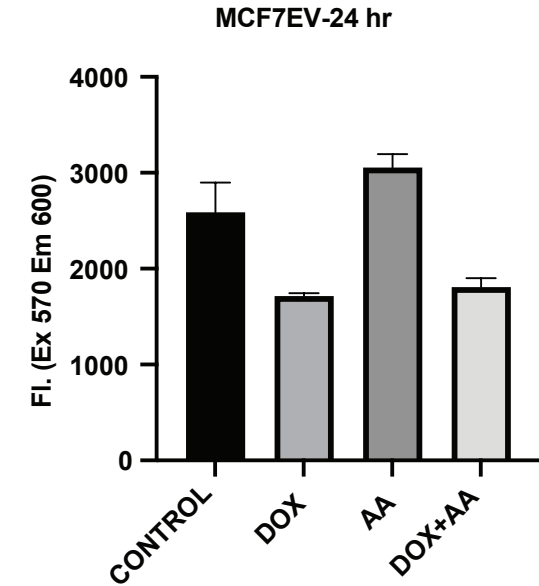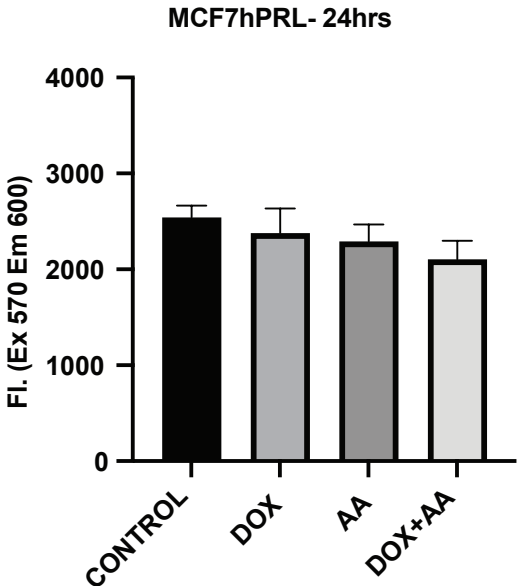

E

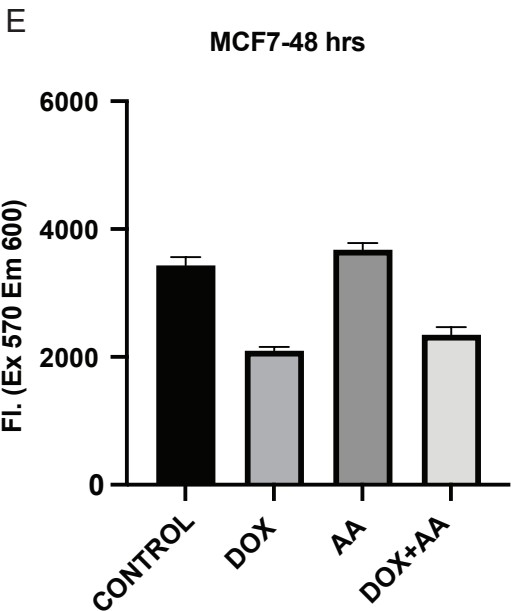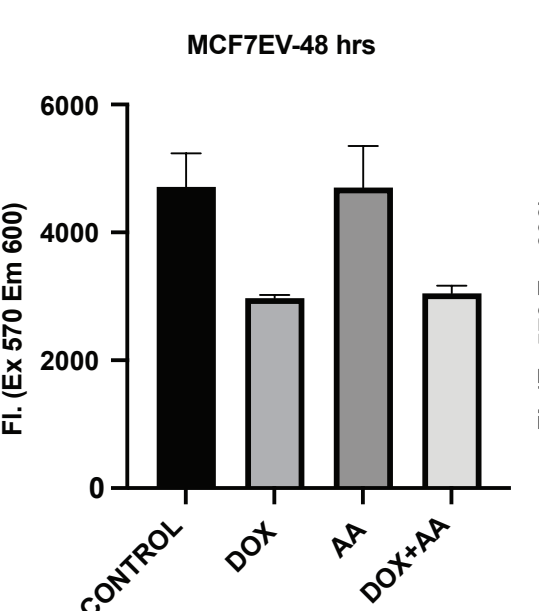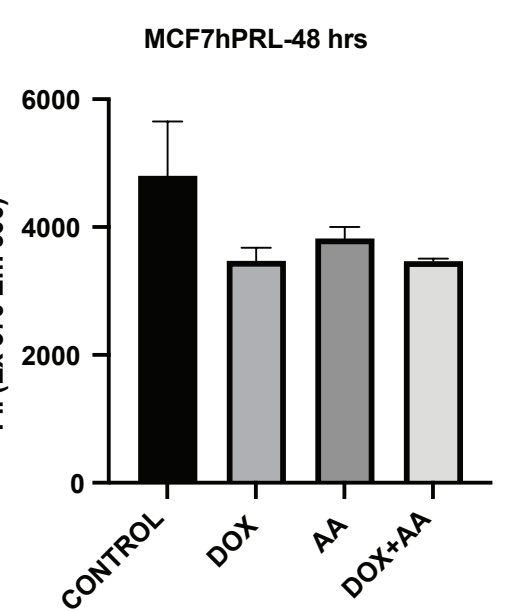

F

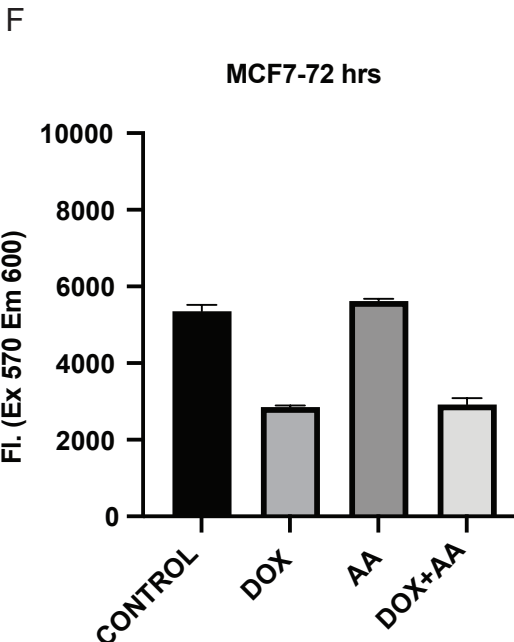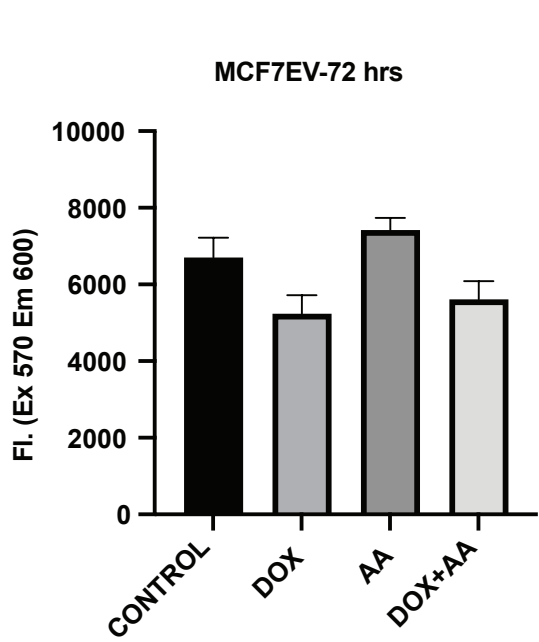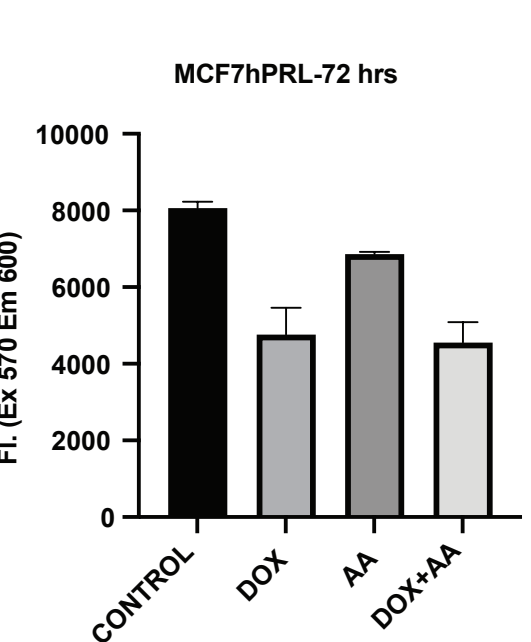

Supplement: Supplementary file 4 [file DataSheet4.pdf]

A

MCF7 1:1 ratio

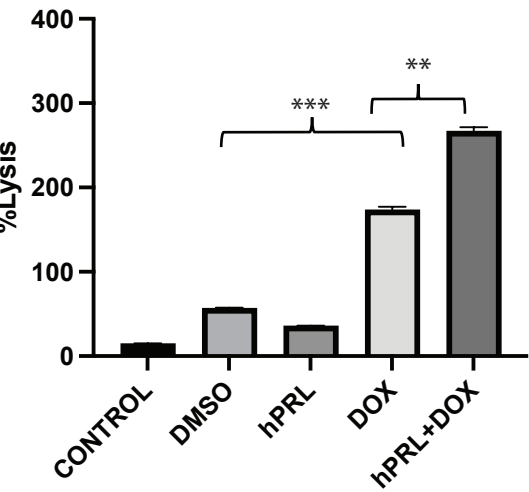

B

MCF7EV 1:1 ratio

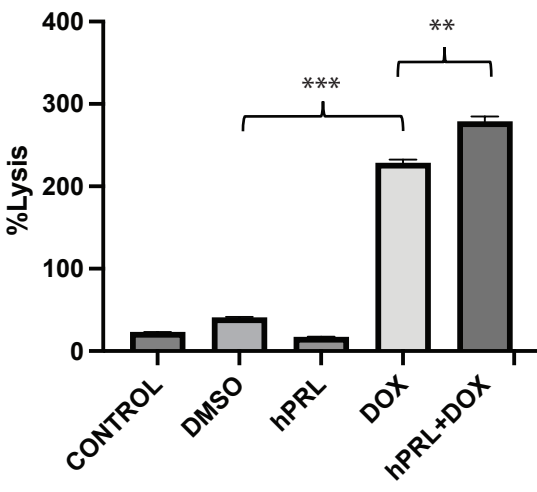

C

MCF7hPRL 1:1 ratio

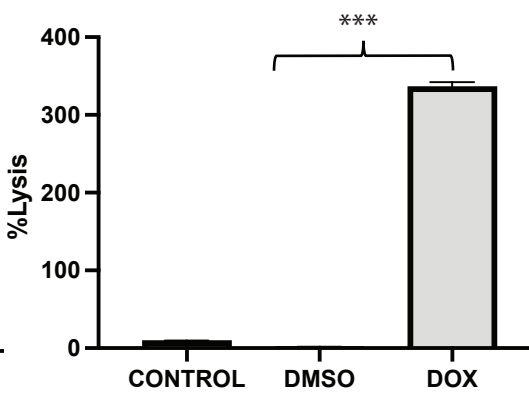

D

MCF7 10:1 ratio

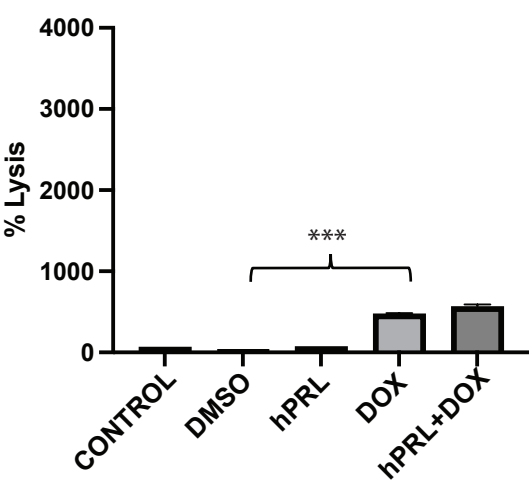

E

SKBR3 10:1

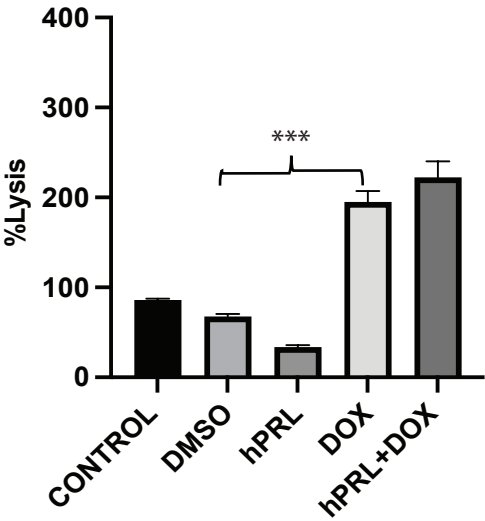

Supplement: Supplementary file 5 [file DataSheet5.pdf]
