# Supplementary material for: Prolactin and DNA damage trigger an anti-breast cancer cell immune response
Source: Front Endocrinol (Lausanne). 2025 Sep 23;16:1586062. doi: 10.3389/fendo.2025.1586062 (PMC12500456; doi:10.3389/fendo.2025.1586062)
Supplement: Supplementary file 6 [file DataSheet6.docx]

Supplementary Material

# Supplementary Materials and Methods

## Immunoblotting for Histone-H3 and PRL

To detect Histone-H3, the PVDF membrane was wet with methanol and blocked in 3% dried skim milk in PBS overnight at 4°C, and washed three times with TBST 0.05%. The membrane was incubated with rabbit anti-H3 primary antibody (Millipore, Billerica, MA, USA) (1:1000 dilution) in 3% dried skim milk in PBS overnight at 4°C, washed and incubated in HRP conjugated goat anti-rabbit secondary antibody (1:10000 dilution) in 3% dried skim milk in PBS for 1 hour before chemiluminescence detection as described (24). To detect PRL, the membrane was blocked in 3% dried skim milk in TBST 0.1% for 2 hours, washed and incubated in monoclonal anti-human PRL antibody (R&D System, Minneapolis, MN, USA) (1:100 dilution) in 5% dried skim milk in TSBT 0.1% overnight at 4°C. The next day the blot was washed and incubated in HRP conjugated goat anti-mouse secondary antibody (1:10000 dilution) in 5% dried skim milk in TBST 0.1% for 30 minutes before washing and chemiluminescence detection.

## Polymerase Chain Reactions

RNA was extracted from breast cancer cells using a Qiagen RNEasy Mini Kit (Qiagen Inc., Mississauga, ON, Canada) according to the manufacturer’s protocol. Complimentary DNA (cDNA) was synthesized from 2 ug of RNA using a Superscript II Reverse Transcriptase kit (Invitrogen) according to the manufacturer’s protocol.

All primers were designed using the NCBI Primer Blast-program. The Operon Oligo Analysis tool was used to detect possible primer dimers and self-complementation was identified with IDT Oligo Analyzer. The desired primers were obtained from the University of Calgary DNA Synthesis Lab (Calgary, AB). Quantitative polymerase chain reactions (qPCR) were carried out using iTaq Universal SYBR Green Supermix (Biorad, Mississauga, ON, Canada) with 1 ml of each forward and reverse primer (final primer concentration of 200 mM) (Table 1). The following protocol was performed for amplification of Sh ble gene: 95 °C for 2 minutes, 40 cycles of 95 °C for 5 seconds, 60 °C for 30 seconds, 78 °C for 20 seconds, and final extension step of 72 °C for 10 minutes, and following protocol was used for amplification of YWHAZ gene: 95 °C for 2 minutes, 40 cycles of 95 °C for 10 seconds, 60 °C for 30 seconds, 78 °C for 20 seconds, and final extension step of 72 °C for 10 minutes in the BioRad MJMini Opticon Real-Time PCR System.

## Conditioned Medium

To investigate secreted PRL levels from human PRL vector transfected MCF7 cells (MCF7hPRL), or empty vector transfected MCF7 cells (MCF7EV), conditioned media (CM) was removed at indicated time points. CM collection was started the day after doxorubicin treatment (day 1) for 7 days. Following centrifugation, secreted protein levels were determined from CM by western blot or human PRL ELISA (Invitrogen, Burlington, ON, Canada), as per manufacturer’s instructions.

## Whole cell extract

10^6^ cells were plated in 10cm plates, and the next day cells were washed with 1X PBS and directly scraped in 1X SDS protein sample buffer, followed by sonication three times for 5 seconds with 5-second intervals at #5 (on dial) (Fisher Scientific 60 Sonic Dismembrator) on ice. Protein samples were snap frozen and stored at -80 °C until use.

## Flow cytometry experimental design

Flow cytometry experiments were optimized with multiple experiments and the final experiments were performed with independent biological replicates. Each mammary gland or each experimental plate was considered as a biological replicate (n).

For the experimental glands that were collected 10 days after cell injection, 3 injected and uninjected mice were used, injected and contralateral glands were processed separately, isolated mammary cells were pooled for n=2 for injected and n=1 for uninjected group and directly used for flow cytometry staining and analysis.

For the anti-asialo GM1 and control serum injection experiments, 6 mice from each group were used to isolate mammary cells, after processing separately, isolated cells were pooled and stained for n=3 for each group.

For *in vitro* studies, 3 independent plates of cells were pre-treated or not with 25 ng/ml human recombinant PRL for 24 hours followed by 2 hours of doxorubicin treatment (1 uM). After 48 hours of recovery, the cells were collected and washed with 1x PBS and used for flow cytometry staining or fixed in 2% PFA after being stained with fixable viability stain. The independent biological replicates were represented as n=3.

## Antibodies

For western blots, the following antibodies were used, anti-Prolactin receptor (D409) Rabbit mAb# 13552, Cell Signaling Technologies, monoclonal anti-human PRL antibody (R&D System, Minneapolis, MN, USA), rabbit anti-STAT5 (phospho Y694) primary antibody [E208] (Abcam), mouse STAT5 primary antibody (Transduction Laboratories, BD BioSciences, San Jose, CA, USA), rabbit anti-H3 primary antibody (Millipore, Billerica, MA, USA), anti-GRB2 (Transduction Laboratories, BD Biosciences, Clone 81), anti-TBP (D5C9H XP® Rabbit mAb #44059.

The following FACS antibodies were used according to the manufacturer’s instructions: BV421 Rat Anti-Mouse F4/80 (BD HorizonTM #565411), CD335 (NKp46) Monoclonal Antibody (29A1.4) PerCP-eFluorTM 710 (eBioscience, 46-3351-82), CD49b (Integrin alpha 2) Monoclonal Antibody (DX5) (eBioscience, 14-5971-85), CD45, HLA-ABC Monoclonal Antibody W6/32 (eBioscience, 14-9983-82), Mouse IgG2a kappa isotype control (eBioscience, 14-4724-82), secondary antibody staining for HLA antibody Donkey anti-mouse IgG (H+L)-FITC (Jackson Immunoresearch, 715-095-151), CD155 Monoclonal Antibody (2H7CD155) FITC (eBioscience, 11-1150-42), mouse IgG1 kappa isotype control FITC (eBioscience, 11-4714-82), CD112 (Nectin-2) Monoclonal Antibody (R2.447) APC (eBioscience, 17-1128-42), Mouse IgG1 kappa isotype control APC (eBioscience, 17-4714-81), MICA/B Monoclonal Antibody (6DE) PE (eBioscience, 12-5788-42), mouse IgG2a kappa isotype control PE (eBioscience, 12-4724,82).

# Supplementary Table 1 Primers

| **Primer name** | **Primers** | **Tm (ºC)** | **Expected amplicon size (pb)** |
| --- | --- | --- | --- |
| Steptoalloteichus hindustanus bleomycin (Sh-ble) (Zeocin resistance gene) | F- 5”-AAGTTGACCAGTGCCGTTCC-3’  R-  5’-CTCCTCGGCCACGAAGTG-3’ | 60°C | 360 |
| Tyrosine 3-monooxygenase/tryptophan 5-monooxygenase activation protein (YWHAZ) | F- 5’-AGTCGTACAAAGACAGCACGTAA-3’  R- 5’-AGGCAGACAAAGGTTGGAAGG-3’ | 60°C | 138 |

# Supplementary Figure Legends

**S1. Confirmation of PRL-secreting and EV-control colonies (A).** PRL levels were measured from whole cell extracts of MCF7 parental cells and MCF7hPRL colonies. GRB2 was used as a loading control. **(B).** Western analysis of PRL secretion from MCF7hPRL colonies (1 to 5) compared with human recombinant PRL standard. **(C).** PCR data showing Sh ble zeocin resistance gene from MCF7hPRL and MCF7EV colonies. YWHAZ was used as a house-keeping gene. **(D).** ELISA analysis of PRL secretion from MCF7hPRL, MCF7EV and parental MCF7 cells over 7 days. The conditioned media collection was started the day after plating the cells (1 x 10^6^ cells/10 cm plate). n=3 for each group. **(E).** p-STAT5 and total STAT5 and histone-H3 (loading control) protein levels from untreated MCF7hPRL cells, or parental MCF7 and MCF7 control EV cells treated with increasing concentrations of human recombinant PRL. **(F).** PRLR and GRB2 (loading control) protein levels from untreated and doxorubicin (1 uM- 2hrs) treated MCF7hPRL, MCF7EV and MCF7 cells. **(G).** Cell viability (Alamar blue) assay showing autocrine PRL-mediated resistance to doxorubicin treatment in MCF7 Cells. Cells were treated with doxorubicin for 2 hrs, then recovered 48 h. The viability of vehicle control (DMSO) treated cells was set to 100%. Graphs represent pooled experiments, n=12. Student t test was used for statistical analysis. Statistically significant analysis (*) denotes *P*<.05, (**) denotes *P*<.01. (Statistical differences for indicated DOX concentrations are, 3 uM (*P*=.006), 4 uM (*P*=.002), 5 uM (*P*=.04), 6 uM (*P*=.003).

**S2. Autocrine PRL delays tumour latency in the presence of DNA damage in SCID mice.** **(A).** Tumour latency in SCID mice after injection of 250,000 MCF7 or MCF7hPRL cells -/+ doxorubicin over 120 days. The sample size is n=5 mice for each group. Log-rank (Mantel-Cox) and Gehan-Breslow Wilcoxon tests were used for statistical analysis.

**S3. Cell viability (Alamar blue) assay showing anti-asialo GM1 does not affect the viability of MCF7, MCF7EV and MCF7hPRL cells in the presence and absence of doxorubicin**. Cells were treated with 20 ul/2.5 ml, 20 ul/2 ml, 20 ul/1.5ml, 20 ul/1.25 ml of anti-asialo GM1. The cell viability was followed for 96 h. Graphs represent pooled experiments, n=6. **(A).** Cell viability in MCF7, MCF7EV and MCF7hPRL cells with anti-asialo GM1 treatment after 24 h, or **(B).** after 48 h, or **(C).** after 72 h **(D).** Cell viability in MCF7, MCF7EV and MCF7hPRL cells with/without doxorubicin (1 uM) and anti-asialo GM1 treatment after 24 h, or **(E).** after 48 h, or **(F).** after 72 h.

**S4. Calcein-AM assay determining the NK cell-mediated lysis of MCF7, MCF7hPRL and MC7EV cells in the presence or absence of DNA damage.** MCF7 and MCF7EV cells were pre-treated with human recombinant prolactin (25 ng/ml) for 24 hours, followed by 2 hours of doxorubicin treatment (1 uM). Cells were trypsinized after 48 hours of recovery time and co-cultured with NK cells in a 1:1 or 10:1 effector/ target ratio. The cell viability of breast cancer cells was determined by Calcein-AM assay and the % Lysis was calculated. **(A).** MCF7 (1:1 E/F ratio). **(B).** MCF7EV (1:1 E/F ratio). **(C).** MCFhPRL (1:1 E/F ratio). **(D).** MCF7 (10:1 E/F ratio). **(E).** SKBR3 (10:1 E/F ratio) Statistically significant analysis (*) denotes P<.05, (**) denotes P<.01, (***) denotes P<.001.
